# Polymeric Powders for Powder Bed Fusion: From Chemistry and Powder Characteristics to Process Parameters, Defects and Applications

**DOI:** 10.3390/polym18050622

**Published:** 2026-03-02

**Authors:** Sina Zinatlou Ajabshir, Helia Mohammadkamal, Zahra Zinatlou Ajabshir, Diego Barletta, Fabrizia Caiazzo, Massimo Poletto

**Affiliations:** 1 Department of Industrial Engineering, University of Salerno, Via Giovanni Paolo II, 132, 84084 Fisciano, SA, Italy; 2 Department of Chemistry, Institute for Advanced Studies in Basic Sciences (IASBS), Zanjan 45137-66731, Iran

**Keywords:** additive manufacturing, powder ved fusion, selective laser sintering, polymeric powders, powder properties, powder characterisation, powder flow behaviour, powder spreading

## Abstract

Polymer powder bed fusion (PBF) is strongly influenced by powder chemistry and powder state, yet many studies discuss the materials and processing conditions in isolation. This review synthesises the literature using a powder-centred framework that connects polymer chemistry and powder production history to measurable powder descriptors, and then links these descriptors to processing windows, defect mechanisms, and application outcomes. Key descriptors include crystallinity and thermal transitions, additive packages, particle size distribution, morphology, and surface texture. Environmental sensitivities are also considered, including moisture uptake, temperature effects, and optical response. These factors are related to powder spreading, energy absorption, and melt solidification or sintering to explain how flowability, packing density, and melt dynamics govern porosity, lack of fusion, distortion, and degradation. Powder qualification is discussed together with lot-to-lot variability and lifecycle effects, including ageing, reuse, and refresh, using the indicators commonly reported in laboratory and production settings and supported by emerging in situ monitoring. Application case studies are consolidated to illustrate how powder state and process control translate into repeatable qualification targets as polymer PBF moves toward a predictable and transferable manufacturing practice.

## 1. Introduction

Powder bed-based additive manufacturing (AM) with a laser beam as the heat source is a 3D-printing route that can be applied to a wide range of materials and is generally referred to as powder bed fusion (PBF). For polymeric materials, this process was originally introduced under the name selective laser sintering (SLS), with Carl R. Deckard credited as the first to develop it [1,2,3]. Although the term “sintering” has persisted, it is largely historical and not strictly accurate, as most current systems operate with partial or complete melting of the polymer rather than purely solid-state sintering [4]. According to ISO/ASTM 52900 [4], the laser-based PBF of polymers is now formally designated as PBF-LB/P. However, for consistency and simplicity, the term PBF will be used throughout this review when referring to the laser-based powder bed processing of polymeric powders.

PBF has reshaped polymer manufacturing by converting loose particles into parts with complex geometry and stable mechanical performance [5]. In this process, a thin powder layer is spread, selected regions are fused by energy input, and the cycle is repeated, enabling tooling-free fabrication with limited post-processing [6,7]. Process stability depends strongly on the powder, because the feedstock governs layer formation, heat transfer, neck growth, and the development of defects [8,9,10]. Build failures are therefore often linked to powder condition and provenance rather than to laser settings alone [9,10]. 

Polymeric powders differ from metal powders in crucial ways. They are softer, more insulating, and more sensitive to moisture and oxygen [11,12]. They charge easily [13], stick to tools, and remember their thermal history [14]. Semi-crystalline polymers exhibit a narrow processing window between the onset of crystallisation and the melting point [15]. Amorphous and low-crystallinity grades behave differently, but they also demand tight temperature control to avoid distortion or incomplete fusion [16]. These traits shift attention from the pure energy input to the balance among chemistry, morphology, and thermal management. In polymer PBF, the powder is not only a carrier of matter, it is an active medium that stores and releases heat, guides crystallisation, and sets the limits of dimensional stability.

The powder production route strongly defines the “identity” of each particle and, in turn, its behaviour in PBF. Depending on how the powder is generated, particles may differ in shape, size distribution, surface texture, internal porosity, crystallinity, and residual contaminants [17,18,19]. These features directly influence flowability, packing density, thermal conductivity, and sintering behaviour, and therefore control how the powder spreads, melts, and bonds during processing [20,21,22]. In practice, the fingerprints created during production travel from the hopper to the spreading tool and are ultimately recorded in the microstructure and performance of the final part [23]. Flow and spreading provide the practical link between particle properties and powder bed quality. When adequate flowability is present, gaps are filled and layers are levelled more uniformly, whereas poor flow can promote caking, arching, and streak formation under the spreading tool. However, high flowability alone should not be taken as a guarantee of good spreadability [24,25]. This behaviour is governed by cohesive forces [26], such as electrostatic charging [27], humidity [28], and temperature gradients [29,30], as well as by particle size distribution (PSD) [31] and shape [32]: fine particles increase surface area and cohesion, while coarse or overly narrow PSDs can reduce packing uniformity; high sphericity lowers interlocking and friction but may also decrease packing density if not balanced by an appropriate size spread [33]. To quantify these effects, flow and spreadability are increasingly assessed using practical powder tests [34]. However, because spreading in PBF is a dynamic event influenced by shear and mechanical disturbances, benchtop results should be complemented by in situ monitoring and machine-level observations to reliably evaluate layer formation [25,35,36,37].

Energy absorption and heat flow connect polymer powder properties to process outcomes in PBF. Compared with metals, polymers show markedly different infrared absorption and heat transfer, and the effective absorption depth is often adjusted through tailored additives [38,39,40]. During processing, laser power, scan speed, hatch spacing, and layer thickness interact with bed temperature and chamber atmosphere to define the thermal history experienced by each voxel of material [41,42,43]. A sufficient melt fraction must be generated to promote neck formation and interlayer bonding, while overheating must be avoided to limit sagging, pore coalescence, and dimensional loss. Because heat accumulates layer by layer, subsurface temperature fields influence subsequent scan tracks, residual stresses can develop, and porosity may evolve through repeated heating and cooling [44,45]. The resulting microstructure and properties are therefore governed by both the instantaneous laser–powder interaction and the cumulative thermal cycle, which can be examined through tools such as differential scanning calorimetry for melt/crystallisation behaviour and microscopy or diffraction methods for phase content and morphology [46,47,48].

Economic and environmental drivers also favour powder reuse, but polymer powders are not thermally inert [49]. Prolonged exposure to elevated temperatures and oxygen can promote chain scission, crosslinking, and oxidation [50,51], with measurable consequences for viscosity, crystallisation kinetics, colour, and flow behaviour [52]. Moisture cycling further modifies cohesion and spreadability, and the optimal refresh strategy is rarely transferable across the polymers, machines, or thermal set-ups [53,54]. For reliable practice, the powder state should be monitored rather than assumed, using fast indicators such as melt flow behaviour, rheological signatures, shifts in thermal transitions, and spectroscopic ageing markers [55]. When reuse, refresh, and sustainability are treated together, material waste can be reduced without sacrificing process stability, provided that the powder life is managed through measurable thresholds and traceable handling [56].

Despite rapid industrial adoption, standards and metrology still lag behind practical needs. Datasheets typically report melting points and moisture limits, yet variables that control spreading, bed uniformity, optical absorption, and interlayer bonding are rarely specified in a comparable way. Cross-platform qualification is further complicated by differences in heating architecture, optics, and gas flow. Harmonised powder descriptors that capture distribution tails in particle size, shape, and roughness, together with surface energy, moisture sorption, and flow functions under relevant humidity and temperature, are therefore essential to connect powder identity to print outcomes. At the same time, emerging formulations, including blends, copolymers, reactive systems, and functional additives, expand capability but also increase the number of coupled variables, making physics-informed and data-driven approaches more attractive when supported by high-quality datasets. 

Several prior review papers have discussed polymer AM in a broad sense, spanning multiple 3D printing routes rather than focusing specifically on PBF [57,58]. Reviews dedicated to polymer PBF have often treated one axis at a time, such as process fundamentals and parameters [59] or consolidation mechanisms and quality control [60]. The distinctive contribution here is the integration of these viewpoints into one powder-centred logic that follows a single causal chain: polymer chemistry and powder production history determine measurable powder descriptors, which shape the processing window and defect mechanisms, and ultimately control part performance. This structure enables consistent cross-study comparisons and provides an integrated synthesis for selecting materials, defining what to measure, and interpreting failure modes during research and qualification. 

This review is structured to follow this chain from polymer chemistry and powder history to descriptors, processing windows, defects, and lifecycle effects. This review addresses that gap by consolidating and organising the evidence across the full chain, linking polymer chemistry and powder history to measurable descriptors, then relating these descriptors to processing windows and mechanism-based defect tendencies under realistic powder spreading and thermal conditions. Rather than prescribing universal settings, the manuscript offers a structured reference of widely used polymer families, their recurring behaviours and sensitivities, and the reporting elements most often needed to compare results across platforms. The aim is to provide a clear starting point for designing experiments, interpreting build outcomes, and accelerating qualification as polymer PBF moves from empirical tuning toward predictable and transferable practice. 

**Figure 1:**
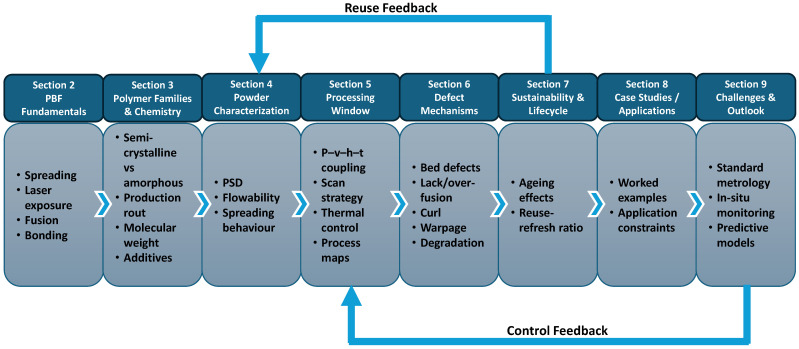
Roadmap of the review. Flow chart showing how the manuscript progresses from PBF fundamentals (Section 2) to polymer chemistry (Section 3), powder characterisation (Section 4), processing windows (Section 5), defect mechanisms (Section 6), sustainability and lifecycle (Section 7), case studies (Section 8), and challenges and outlook (Section 9), with feedback loops for reuse and process control.

This paper is organised as follows: Section 2 outlines polymer PBF fundamentals, while Section 3 and Section 4 cover polymer families, chemistry, powder production routes, and the powder descriptors used for comparison, Section 5 and Section 6 then connect these descriptors to processing windows and mechanism-based defect pathways, and Section 7 discusses sustainability and lifecycle aspects with an emphasis on powder ageing, reuse, and refresh practice. Application case studies are presented in Section 8, followed by future challenges and outlook in Section 9. Figure 1 provides a roadmap of the review and highlights the powder-to-process-to-defect logic used throughout. 

## 2. Powder Bed Fusion Process and Operating Principle

**Figure 2:**
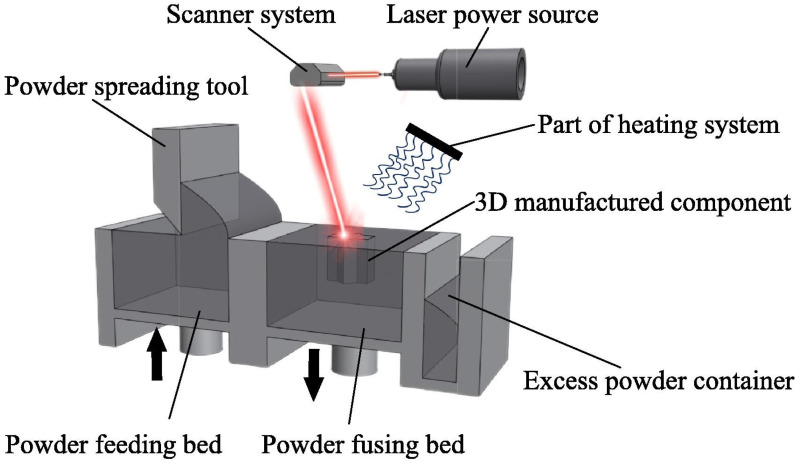
Schematic overview of the PBF process. The black arrows indicate the vertical movement of the build platforms during powder feeding and fusion. Reproduced from [25], Elsevier, 2024.

Additive manufacturing (AM) produces three-dimensional parts directly from digital designs by building them layer by layer [61]. It offers clear advantages over conventional processes, such as forging [62], welding [63], extrusion [64], and casting [65]. Powder-based AM uses free-flowing powders as the starting material and creates components in a layerwise manner by selectively bonding only the regions defined by the digital design. Within this family of processes, powder bed fusion (PBF) has emerged as the reference technology for producing high-performance engineering parts with complex geometries. Figure 2 shows an overall overview of the PBF process.

PBF is a thermal AM process [66] where a thin layer of powder is spread across a build platform and selectively fused using a focused energy source, such as a laser beam (PBF-LB) [4,67] or electron beam (PBF-EB) [4,68]. The powder spreading step [25,69,70] is also called powder recoating [71,72]. Less commonly, it is referred to as powder paving [73] or powder delivery [74]. After each layer is fused, the platform lowers and a new powder layer is applied. This continues until the full part is built. PBF requires powders that are spherical, and effectively flowable [75]. These characteristics ensure uniform powder spreading and packing. The typical particle size ranges from 15 to 200 µm for various materials [76]. Inadequate powder quality can lead to poor layer formation, lack of fusion, porosity, and microstructural defects. Due to the rapid melting and solidification, powder behaviour also affects microstructural defects [77,78], residual stresses [79,80,81], and mechanical anisotropy in the final part [82]. In polymer PBF, the part quality is governed not only by laser exposure but by thermal field stability and spreading physics. Preheating the bed close to the onset of melting reduces the additional laser energy required for coalescence, but an excessive bed temperature can promote unwanted sintering during spreading and polymer degradation during long dwell times. The scan strategy (e.g., alternating hatch directions, island strategies, contour timing) controls heat accumulation and residual stress, while gas flow and low oxygen partial pressure are essential for oxygen-sensitive polymers during long builds. The layer thickness must be selected together with the powder size distribution: the effective layer thickness (spreading gap) should exceed the coarsest particles, typically on the order of ~2–3 d_90_, to reduce gouging, jamming, and streak-driven density variations that later appear as porosity or lack-of-fusion regions.

**Figure 3:**
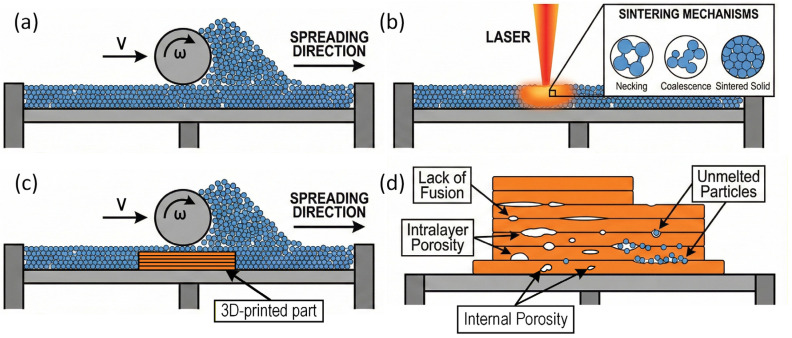
Schematic of the PBF process for polymeric powders: (**a**) a thin powder layer is spread on the build platform using a roller; (**b**) a laser selectively heats and sinters the polymeric powder; (**c**) a new powder layer is spread over the previously sintered layers and the printed part, and the process is repeated layer by layer; (**d**) representative defects that may form during the PBF of polymeric powders.

In the PBF of polymeric powders, the critical step is the laser-induced sintering of partially molten particles rather than simple melting of a bulk pool. When the laser passes over the preheated bed, only a thin surface region of each polymer particle is driven into a viscous or molten state, so that neighbouring particles form necks and gradually coalesce under the action of surface tension. Continued exposure and bed heating promote both intralayer consolidation and bonding with the previously processed layer, while the subsequent cooling step freezes in the final crystallinity and residual porosity. Insufficient or non-uniform energy input during this sequence can lead to lack of fusion, intralayer pores, and islands of unmelted powder. Figure 3 schematically illustrates these powder spreading and sintering mechanisms and the associated defects that can develop during the PBF of polymeric powders. 

Polymer PBF is governed by a simple but strict chain of causality. Everything begins with the powder, because the powder is both the feedstock and the medium that must spread, absorb energy, and fuse repeatedly under a controlled thermal field. For this reason, the discussion in this review is intentionally organised around features that carry through that chain and remain predictive of build success. Powder production history is considered where it leaves a measurable signature on particle size tails, morphology, surface texture, and chemistry, because these factors control flow, packing, and layer uniformity. The process description then focuses on the parameters that directly interact with this powder state, such as layer formation conditions, thermal management, and energy delivery, because they define the processing window and the dominant defect pathways. Applications are discussed where they expose clear constraints on repeatability, performance, and qualification, and therefore help translate mechanisms into practical targets. This scope avoids repeating datasheet-level information and instead follows the natural logic of PBF: powder identity and behaviour shape process stability, which shapes defects and microstructure, and ultimately determines part properties and functional outcomes.

## 3. Materials and Powders

In polymer PBF, the powder is the enabling medium that turns digital designs into functional parts across industries. Available powders now span soft, elastic grades for wearables and cushioning, durable general-purpose options for consumer and automotive components, medical-oriented materials for patient-specific devices, and high temperature families for aerospace and under-hood environments. The chosen powder defines the feasible processing window and the attainable balance of strength, toughness, heat resistance, and surface finish. As this portfolio expands and standardises, polymer PBF moves from prototyping toward reliable, application-ready manufacturing. 

### 3.1. Polymer Families for PBF and 3D Printing 

The polymer families covered here represent the main material space used or actively developed for laser-based polymer PBF. They include dominant commercial feedstocks and high performance or emerging polymers that extend temperature capability, chemical resistance, or functional performance. Both semi-crystalline and amorphous systems are included because they respond differently to the PBF thermal cycle and show different risks in shrinkage, bonding, and dimensional stability.

Semi-crystalline polyamides dominate polymer PBF because they offer a practical processing window and stable properties. Polyamide 12 remains the reference due to its balanced melting and crystallisation behaviour [83,84]. Polyamide 11 is also widely used and is typically tougher, with bio-based feedstock routes [85,86]. Other polyamides are possible but more demanding. Polyamide 6 (PA6, Nylon-6) can achieve good strength and fatigue performance, but moisture uptake and fast crystallisation narrow the usable window [87,88]. Polyamide 1010 can show lower moisture uptake and higher ductility, which can help dimensional stability when suitable powders are available [89].

Beyond polyamides, several families are attractive but often have tighter processing windows. Poly(butylene terephthalate) [90,91,92] and high density polyethylene (HDPE) [18,93] offer chemical resistance and low density, but both are sensitive to thermal control and shrinkage. In practice, they often need careful bed temperature control and improved absorption to reduce lack of fusion, curl, and warpage. Polypropylene (PP) [94,95,96] is also attractive for lightweight functional parts because of low density and chemical resistance, but its shrinkage and melt flow behaviour can increase distortion and porosity unless the powders and thermal settings are well tuned.

High-performance poly(aryletherketones), such as poly(etheretherketone) (PEEK) [97] and poly(etherketoneketone) (PEKK) [98], enable demanding aerospace, medical, and high temperature applications. Their main limitation is the narrow thermal balance between fusion, crystallisation control, residual stress, and degradation. PEEK crystallises quickly and therefore needs tight thermal control to reduce warpage [99] [99]. PEKK crystallises more slowly and its behaviour can be tuned through composition, which can widen the usable window and improve feature retention [100]. Since these polymers often absorb weakly at common laser wavelengths, absorbers or pigments may be needed to stabilise the energy coupling without harming the target properties.

Polymer blends are increasingly used to combine properties or to widen processability, but compatibility and shrinkage mismatch remain major risks. Reported blend systems include PA12/PBT [101], PA12/PEEK [19], PA12/HDPE [102], PBT/polycarbonate (PC) [103], and styrene–ethylene–butylene–styrene/polypropylene (SEBS/PP) [104]. Thermoplastic polyurethane (TPU) [105,106,107] adds elasticity for lattices and flexible parts, but it can show high porosity and thermal sensitivity unless the powder flow and thermal control are improved.

Amorphous polymers occupy a smaller niche and often show higher porosity than semi-crystalline systems. Polycarbonate (PC) can be processed, but dense parts are difficult and porosity can remain high, with regulatory limits for some uses [108]. Polystyrene (PS) can deliver good dimensional accuracy when parameters are well controlled [109]. Polymethyl methacrylate (PMMA) also shows that performance can improve with optimisation, but porosity can still be a major limitation [110]. Styrene–acrylonitrile (SAN) can offer good accuracy but may form internal voids that sometimes require post-processing, such as infiltration [111]. Overall, semi-crystalline polyamides and high performance PAEKs currently offer the most reliable balance between density, stability, and processability, while progress in polyolefins, polyesters, and tailored copolymers is gradually expanding the usable polymer set.

Biopolymer use in PBF spans bio-based and biodegradable systems [112]. PA11 is a key bio-based option for durable parts. Biodegradable polymers, such as polylactic acid (PLA),or ε-Polycaprolactone (ε-PCL), are mainly explored for porous scaffolds and resorbable devices, but they need stricter moisture control and careful thermal management due to narrow windows and ageing sensitivity [113]. In biomedical additive manufacturing more broadly, common synthetic polymers include PLA [114,115,116,117], PCL [118,119], polyethylene glycol (PEG)-based systems [120,121], polyglycolic acid (PGA) [122,123], poly(propylene fumarate) (PPF) [124], and thermoplastic polyurethanes (TPU) [106,125]. Other biocompatible options are reported for specific needs, including poly(amino acids) (PAAs) [126], used mainly in other printing processes [127], poly(butylene succinate) (PBS) [128,129], polydimethylsiloxane (PDMS) [130,131], and surface eroding polymers, such as polyanhydrides (PA) [132,133] and poly(ortho esters) (POE) [134,135], that are attractive for drug release but difficult to process. Additional candidates include polydioxanone (PDX)- and polyphosphazene (PPHOSs)-based systems, often via blends to improve printability [136]. These materials highlight the direction of application-driven powder design, but PBF adoption remains strongest where powders can be engineered for stable powder spreading, controlled fusion, and predictable degradation behaviour.

Chemical architecture governs both fusion and solidification during cooling [137]. The molecular weight distribution sets the melt viscosity, while stabilisers and chain end chemistry affect sensitivity to oxidation and chain scission [138,139]. Semi-crystalline polymers melt and then crystallise during cooling, and crystallinity controls the modulus and shrinkage. Cooling rate and nucleation conditions shape crystallite size and therefore dimensional stability and residual stress risk [137,140,141]. Amorphous systems avoid crystallisation shrinkage but can show long relaxation times and creep if thermal history is not controlled [142,143]. Fillers and absorbers also change optical response and can shift the crystallisation behaviour. Oxygen and moisture scavengers can reduce degradation during long builds and repeated reuse [144].

**Figure 4:**
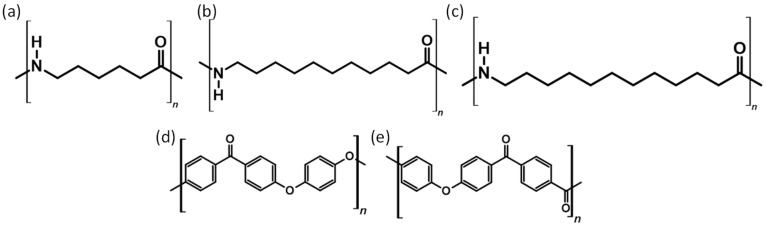
Repeating chemical units of common polymers used in PBF: (**a**) PA6, (**b**) PA11, (**c**) PA12, (**d**) PEEK, and (**e**) PEKK.

Figure 4 shows the chemical structure of some of the famous polymers used in PBF.

### 3.2. Polymer Chemistry and Additives

Polymer chemistry and additive packages strongly control how polymer powders behave during the PBF thermal cycle. Molecular weight and its distribution set melt viscosity and chain mobility. This directly affects neck growth, interlayer bonding, and dimensional stability. Additives such as stabilisers and optical absorbers can change energy absorption, ageing resistance, and powder flow. As a result, the usable process window and defect sensitivity can shift even when the polymer family name stays the same.

A clear example is polypropylene, where a small chemistry change can reshape both its processability and properties. A comparison between isotactic polypropylene and a polypropylene ethylene random copolymer shows this effect. Under similar powder morphology and processing conditions, isotactic polypropylene had a wide sintering window of about 26.3 °C, but poor flow and high porosity produced brittle parts with about 14.9 MPa tensile strength and about 1% elongation. The random copolymer had a very narrow sintering window of only 2.2 °C, so temperature control became more critical. However, it produced tougher parts with about a 19.5 MPa tensile strength and about 207% elongation, and it promoted a dominant gamma crystalline phase not seen in injection moulded counterparts. This confirms that small changes in macromolecular structure can strongly shift the process window, crystallisation pathway, and achievable property space in polymer PBF [46].

**Figure 5:**
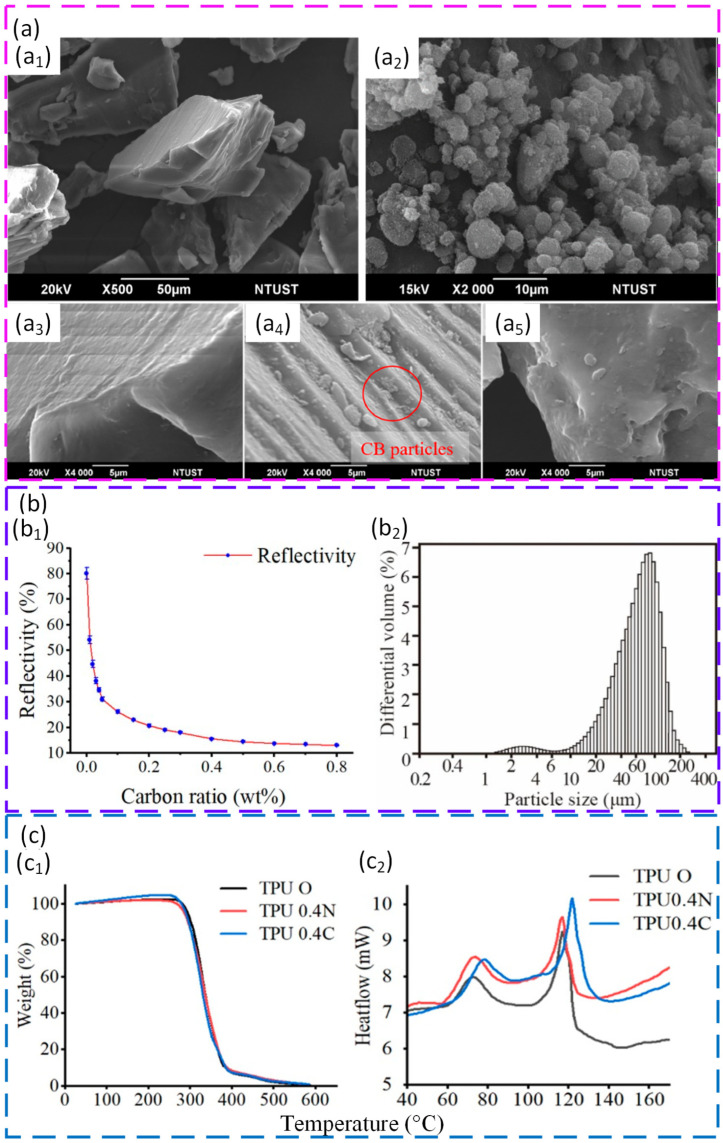
Effect of carbon black coating on TPU elastomer powder: (**a**) powders and morphology, including (**a1**) TPU powder, (**a2**) carbon black, and magnified views of (**a3**) virgin TPU (TPU 0), (**a4**) TPU mixed with carbon black at room temperature (TPU 0.4N), and (**a5**) TPU mixed with carbon black with heating at 70 °C (TPU 0.4C); (**b**) powder characteristics including (**b1**) reflectivity versus carbon black content and (**b2**) particle size distribution; (**c**) thermal behaviour by (**c1**) TGA and (**c2**) DSC. Reproduced from [125], MDPI, 2024.

Surface engineering can also be used to tune laser coupling, but it has an optimum. Carbon black coating of thermoplastic polyurethane illustrated this point. Coating at about 70 °C enabled uniform adhesion without premature fusion and produced a narrow particle size distribution suitable for PBF. Reflectivity dropped from roughly 80% for virgin TPU to about 14% at an optimal carbon black level of 0.4 wt%. Higher carbon black levels reduced reflectivity further, but they started to reduce interparticle bonding by limiting polymer–polymer contact. Under optimised PBF conditions, the 0.4 wt% coated powder produced parts with tensile strength of about 7.9 MPa, elongation at break of about 365%, hardness of 78 A, and density of 1.09 g cm^−3^. Micro-CT also showed fewer and smaller pores than commercial TPU powders. Overall, this case shows a practical design rule. Absorption can be improved by surface additives, but excessive loading can reduce bonding and harm density [125]. Figure 5 shows how carbon black coating affects TPU powder morphology, reflectivity, particle size, and thermal behaviour compared with virgin TPU.

**Figure 6:**
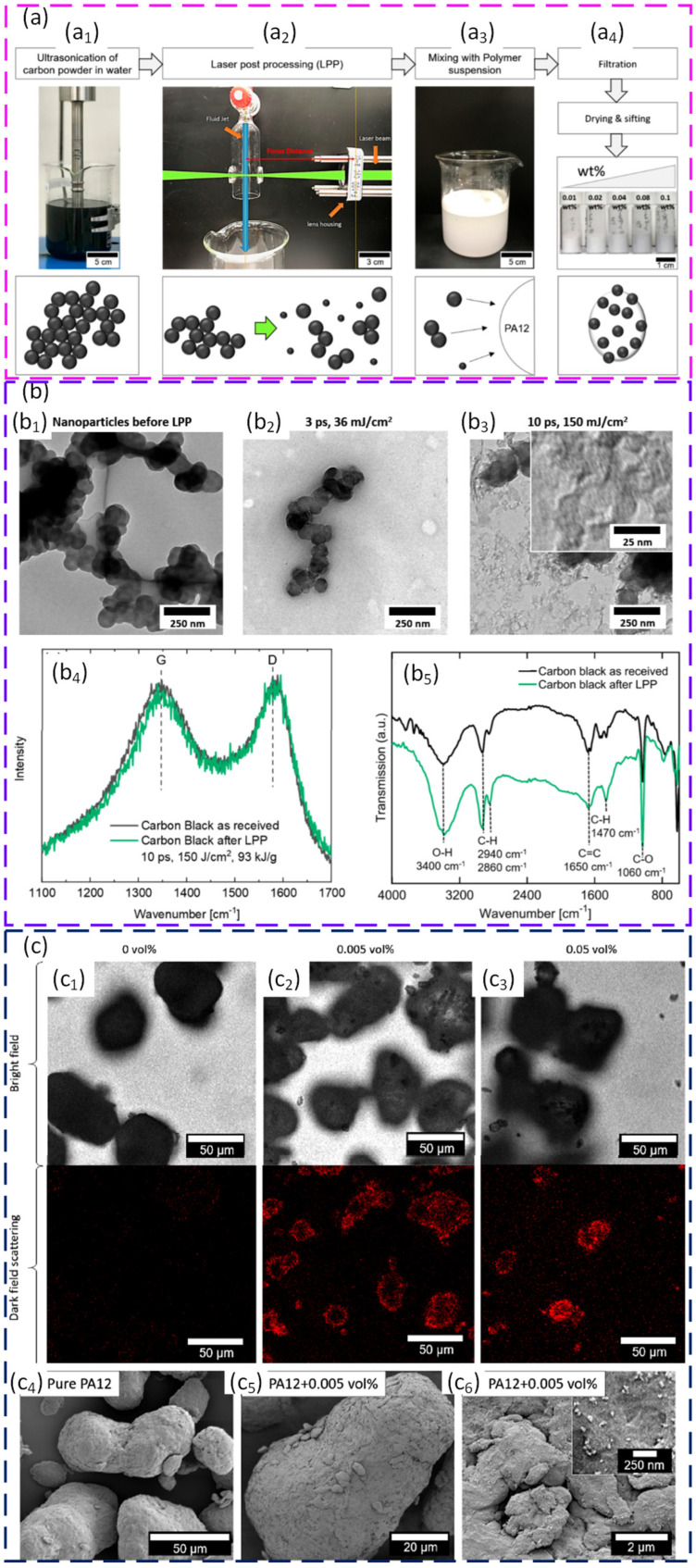
Colloidal carbon nanoparticle additivation of PA12: (**a**) process chain, including (**a1**) ultrasonication dispersion, (**a2**) laser post-processing (LPP) in a liquid jet, (**a3**) mixing with PA12 for adsorption, and (**a4**) filtration, drying, and sieving; (**b**) nanoparticle characterisation, including (**b1**) TEM of raw nanoparticles, (**b2**,**b3**) colloids after LPP at 36 and 150 mJ/cm^2^, (**b4**) Raman spectra, and (**b5**) FTIR spectra before/after irradiation; (**c**) nanoparticle dispersion on PA12, including confocal images of (**c1**) pure PA12 and PA12 with (**c2**) 0.005 and (**c3**) 0.05 vol% nanoparticles, and SEM images of (**c4**) unmodified and (**c5**,**c6**) additivated PA12 showing homogeneous surface coverage. Reproduced from [145], MDPI, 2020.

Nanoscale additives can modify crystallisation and microstructure, but dispersion quality is often more important than the additive chemistry itself. For PA12, laser synthesised carbon nanoparticles deposited colloidally at very low loadings down to 0.005 vol% shifted crystallisation to higher temperatures and produced finer, more oval lamellar structures. Powder flow was maintained, so the practical window could be used more reliably. A follow-up work showed that well-dispersed nanoparticles introduced through aqueous colloidal routes preserved the free-flowing behaviour and improved ductility at constant strength. By contrast, the same nanoparticles, or silver nanoparticles, applied by dry coating reduced flow, hindered interlayer diffusion, and led to larger irregular pores and lower density, even though the gamma phase of PA12 was retained. The main message is that surface distribution controls the balance between crystallisation control and defect risk in PBF [145,146]. Figure 6 illustrates the colloidal additivation route used to decorate PA12 powder with carbon nanoparticles and shows that, after laser post-processing, the nanoparticles are structurally modified and homogeneously distributed on the PA12 particle surfaces.

Polymer chemistry can also be tuned without external additives by using molecular design as an internal modifier. In a study using polypropylene, blends of very low molecular weight powder (12k) with high molecular weight grades (250 k and 340 k) reduced zero shear viscosity enough to accelerate neck growth within the short thermal dwell typical of PBF. Comparable coalescence was achieved in seconds rather than tens of seconds, and the low molecular weight fraction remained distributed in the melt rather than forming a surface layer. When printed, these blended powders produced parts with less void space, higher crystallinity, and large stiffness gains, with storage modulus increases approaching 200% compared with unimodal high molecular weight powders. At the same time, a clear lower limit appeared. Neat 12k powder became too fluid and over coalesced, so geometric fidelity was lost. This shows that molecular weight blending can widen the effective window, but rheological limits still define printability [147,148].

Molecular weight, reflected in chain length and its distribution, sets the mobility time scale of polymer segments and therefore strongly shapes the heat treatment needs in polymer PBF. Longer chains increase entanglement density and melt viscosity, slowing stress relaxation and interdiffusion across fused particle interfaces; effective thermal conditioning then tends to require longer dwell times and carefully selected temperatures that remain below degradation limits. Shorter chains relax and flow more readily, but they can be more sensitive to oxidative chain scission and may exhibit different crystallisation responses because the balance between nucleation and growth shifts with mobility. During heating, semi-crystalline powders evolve from a two-phase solid, where crystalline lamellae are embedded in an amorphous matrix, to a softened state as segmental motion increases, and then to a melt as crystals disappear over a temperature range rather than at a single point. During cooling, crystallisation reappears by nucleation and growth, and the cooling rate controls how much ordering can develop, affecting crystallinity level, lamellar thickness, and spherulite size, with direct consequences for shrinkage and residual stress. In this context, Avrami-type kinetics offer a practical way to describe the time evolution of the crystallised fraction during approximate isothermal holds, because they capture the combined effect of nucleation and growth in a compact form; however, they should be treated as an approximation and are expected to lose accuracy under strong non-isothermal histories, secondary crystallisation, or heterogeneous, transport-limited conditions.

### 3.3. Powder Production Routes and Their Signatures

The production route leaves clear signatures in particle size distribution, sphericity, surface condition, and internal defects [149]. This section focuses on route effects that matter directly for polymer PBF. Resin synthesis is only discussed when it changes powder performance through residual volatiles, molecular weight shifts, or surface chemistry that affects flow, absorption, or degradation. The emphasis is on routes that can reach PBF relevant size ranges, provide morphology control, and are described with enough detail to connect production history to measurable powder descriptors. A practical interpretation is that production history is a primary lever for stable powder spreading, consistent laser coupling, and reduced defect risk during printing.

#### 3.3.1. Milling and Grinding

Mechanical size reduction methods, such as milling and grinding, can tune the mean size and the width of the size distribution, but they often produce irregular particles and rough surfaces. Cryogenic milling increases brittleness and limits thermal degradation, yet it typically yields angular particles with broader distributions and higher cohesion. Classification and surface conditioning are often needed to reach reliable powder spreading.

Wet stirred media milling can be an energy efficient alternative to cryogenic impact milling when temperature and solvent properties are controlled. For polystyrene (PS) and polyetheretherketone (PEEK), a lower grinding temperature increased brittleness and improved comminution, but solvent viscosity created a practical barrier at very low temperatures. Switching from ethanol to n hexane reduced viscosity and enabled finer PEEK powders at a low temperature, showing that solvent viscosity can limit milling efficiency as much as polymer toughness [150].

Dry coating can partly compensate for the weak spreading performance of cryogenically ground powders. In a study using cryogenically ground polyamide 12 (PA12), adding small amounts of hydrophobic silica (SiO_2_) or carbon black (CB) improved flow, but the two additives affected PBF differently. SiO_2_ improved flow but did not increase absorption, so fusion remained poor and porosity stayed high. CB improved flow and also increased energy uptake, which reduced porosity and improved tensile properties. CB also suppressed electrostatic charging and reduced powder sticking to the powder spreader [151]. This comparison shows a consistent trend. Flow aids that also stabilise optical coupling are more likely to improve both spreadability and part density.

Cryogenic jet-based micronisation can support continuous classification and controlled temperature operation, but reaching very fine sizes remains challenging for some polymers. Even with stable low temperature operation, sizes below about 40 µm can remain difficult to achieve, which sets a practical limit for some jet-based systems [152].

Very fine micro- and nanoscale fragments can be produced by cryogenic ball milling, as shown in microplastics and nanoplastics studies. These works are valuable mainly for two reasons. First, size distributions can become strongly material dependent and bimodal. Second, single measurement methods can miss the finest irregular fraction, so combined characterisation is needed when benchmarking powders [153,154]. For PBF feedstocks, the key message is that fine tails must be measured reliably because they strongly affect cohesion and spreading.

Highly filled composite powders can also be produced through combined mechanochemical processing and cryogenic size reduction. For PA12 with hexagonal boron nitride (h BN), solid-state shear milling (S3M) followed by cryogenic pulverisation produced ellipsoidal composite particles with good spreadability when a small amount of silica was used. Thermal conductivity increased substantially with filler loading, while voids reduced the mechanical strength. A post-infiltration treatment could recover mechanical properties, showing the typical trade-off between thermal function and defect sensitivity in highly filled powders [155].

#### 3.3.2. Spray Drying

Spray drying forms particles from droplets. It often produces rounder shapes and narrower distributions than mechanical milling, although hollow particles, shell structures, or internal voids can appear and must be managed [156]. Spray drying is widely used in pharmaceuticals because it provides strong control over particle size, morphology, and flow, including for micropowders and submicron powders [157,158]. For polymer PBF, the main benefit is the ability to engineer near spherical particles with a tailored surface condition, while the main challenge is reaching the desired size window with high yield and limited internal porosity.

For polysulfone (PSU), spray drying produced spherical microspheres with preserved thermal characteristics, but the particle size was below the typical target range for PBF. Rotor milling could produce rounder particles in a PBF relevant size range without significant degradation. High energy ball milling produced very fine angular particles and clear signs of polymer damage, including discoloration and thermal shifts, which indicates unacceptable degradation for PBF feedstock use [159]. This comparison supports a simple guideline. Mild routes can preserve polymer chemistry, while overly aggressive mechanical routes can damage the backbone and narrow usability.

**Figure 7:**
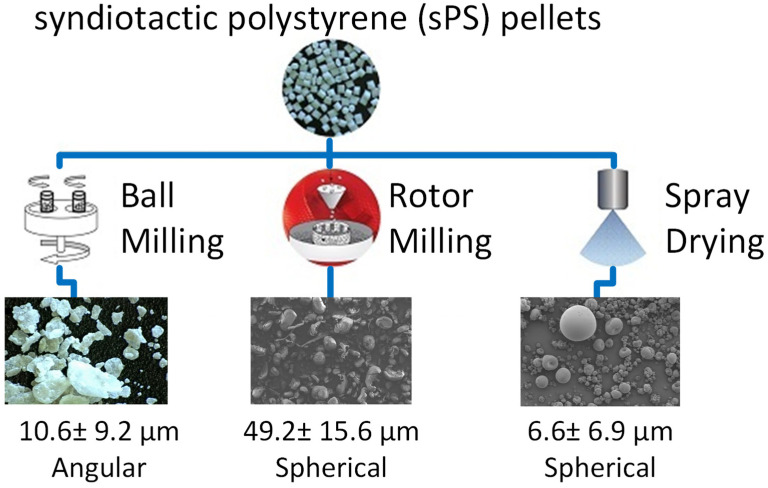
Comparison of polymeric powder production routes for syndiotactic polystyrene used in PBF, highlighting milling versus spray drying and the resulting powder morphologies. Reproduced from [160], MDPI, 2016.

A similar pattern was reported for syndiotactic polystyrene (sPS). Spray drying produced spherical particles with low degradation, but they were too small for PBF, largely due to concentration limits during solution processing. Rotor milling produced rounded particles in the target size range with acceptable flow. High energy ball milling caused severe degradation and a strong loss of crystallinity. Proof of single layer sintering showed good coalescence but also warpage under strong thermal gradients, highlighting the need for robust preheating and thermal control for this polymer family (see Figure 7) [160].

#### 3.3.3. Precipitation and Melt Emulsification Routes

Precipitation routes control size and surface formation through solvent–nonsolvent exchange. They can yield smooth or textured surfaces and can shift crystallinity, porosity, and thermal behaviour depending on the phase separation path. Melt emulsification (ME) forms particles by shearing a molten polymer into droplets and then solidifying them. Emulsion and suspension-based approaches can also yield near spherical powders, but surfactant residues and internal porosity can affect flow and sintering.

**Figure 8:**
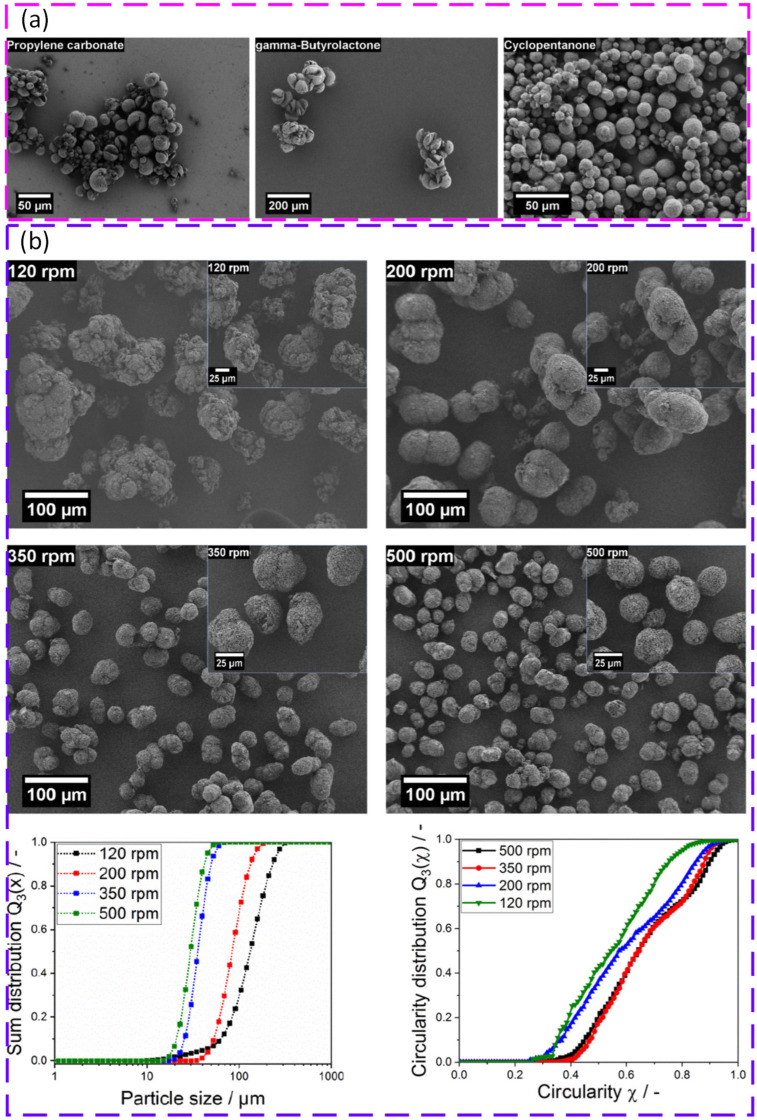
PBT powder production by liquid–liquid phase separation (LLPS): (**a**) influence of solvent selection on the morphology of precipitated PBT particles (SEM images); (**b**) effect of stirring speed (120–500 rpm) during LLPS and crystallisation from cyclopentanone on particle microstructure, size distribution, and circularity. Reproduced from [163], Elsevier, 2021.

Liquid–liquid phase separation (LLPS) has emerged as a coherent route to engineer spherical, free flowing powders for polymer PBF. A central advantage is that particle size, sphericity, and crystallinity can be tuned using solvent choice, concentration, and temperature history. LLPS processing of poly(L lactide) (PLLA) produced spherical powders with narrow distributions, high intrinsic flowability, and a wide sintering window, supporting biodegradable PBF feedstocks without relying on halogenated solvents [161]. The concept was then transferred to polyoxymethylene (POM), where solvent selection and precipitation control enabled spherical, free flowing powders with good spreadability and successful PBF builds without extra flow agents [162]. For polybutylene terephthalate (PBT), LLPS produced near spherical particles with narrow distributions and increased crystallinity. After a simple dry coating step, flowability improved further and stable layer formation at a high temperature was reported, indicating strong promise for engineering polyester powders for PBF [163]. Figure 8 shows how liquid–liquid phase separation conditions, namely solvent choice and stirring speed, control the morphology, size distribution, and circularity of PBT particles produced for PBF. A useful conclusion from these studies is that LLPS can decouple powder shape engineering from polymer family selection, but success depends on solvent compatibility, controlled phase separation, and drying history.

Thermally-induced phase separation (TIPS) is another route to spherical powders, including for polymers that are difficult to process by standard routes. For polypropylene (PP), TIPS enabled spherical powders that were processed into multilayer parts, including from the recycled PP feedstock. Particle size was controlled by concentration, quench temperature, and molecular weight, consistent with droplet coalescence-controlled phase separation. This provides a pathway to expand polymer portfolios while supporting circularity goals [164].

TIPS-based precipitation can also embed functional fillers inside particles rather than relying on surface mixing. For polyamide 11 (PA11), iron oxide additives were entrapped during particle formation, improving thermal stability and maintaining PBF processability while delivering magnetic functionality [165]. A related precipitation-based approach for PBT showed that molar mass can tune melt viscosity and crystallisation kinetics while leaving external powder attributes and flow behaviour largely unchanged. This supports a design strategy where melt behaviour is tuned without sacrificing spreading stability [166].

Melt emulsification is attractive for shaping near-spherical powders with strong flow and packing, but thermal and hydrolytic history can become critical. In one study, ME produced PP particles with sizes relevant to PBF when viscosity was reduced and droplet coalescence was controlled. This demonstrated that ME can be extended beyond waxes to technical polymers [167]. For PBT, an extrusion-based ME route created highly spherical particles with excellent flow after classification, but the thermal exposure and washing steps led to hydrolytic degradation and a narrower sintering window. This caused curling and build instability and a reduced mechanical performance compared with the base polymer [90]. The central message is that ME can deliver ideal morphology, but polymer stability must be protected during processing, washing, and drying.

A combined ME and spray agglomeration strategy can also convert very small primaries into larger spherical agglomerates suitable for powder bed processes. This approach produced highly spherical wax-based powders with improved flow after dry coating and enabled printing on a desktop system. It provides a useful process blueprint for future extension to higher molar mass thermoplastics [168]. 

#### 3.3.4. Advanced Techniques

Advanced routes, such as supercritical processing, atomisation, plasma-based treatments, and engineered immiscible blending, aim to increase sphericity, clean surfaces, and reduce contamination. Cost, throughput, and scale up remain common constraints [169]. In practice, these routes still require classification, controlled drying, and conditioning to manage fines, moisture, and electrostatics before printing. A useful way to group these approaches is by whether they primarily control particle formation during droplet generation, reshape particles after formation, or modify only the surface chemistry.

Supercritical assisted atomisation (SAA) uses supercritical carbon dioxide (SC CO_2_) to expand a polymer solution before atomisation. It can produce near spherical micro scale particles under mild thermal conditions, with the particle size controlled by solution concentration, CO_2_ ratio, and temperature relative to the glass transition temperature [170]. This route is promising for sensitive formulations, though size ranges must be matched to PBF needs. While SAA controls particle formation during atomisation, alternative routes achieve sphericity through melt-mediated shaping driven by interfacial tension.

Immiscible blend-based routes can sculpt spherical powders by forming droplets in a removable matrix. An early work showed that polyamide 12 (PA12) and poly(butylene terephthalate) (PBT) domains can round through interfacial tension in suitable matrices [171]. A controlled melt blending extraction (MBE) approach identified the viscosity ratio as a key design parameter and produced spherical PA12 powders with strong flow and a wide sintering window. This supports a scalable route where rheology, not just chemistry, is used to engineer powder shape and usability [172]. These melt-based approaches mainly reshape particle geometry, whereas plasma treatments target surface chemistry while keeping particle size distribution and morphology largely unchanged.

A plasma-based surface treatment can modify wettability and surface chemistry without changing particle size distribution or morphology. In a study using PA12, PE HD, and PP, an atmospheric pressure plasma jet (APPJ) in a fluidised bed reactor (FBR) produced strong wettability changes with an optimum exposure time. Longer exposure could increase etching and reduce benefits. The method offers a scale up path through reactor design rather than changing the polymer itself [173]. In contrast to low-temperature atmospheric plasma, thermal plasma routes can actively reshape particles and, in composites, improve sphericity and packing while maintaining polymer integrity when the temperature is controlled.

Radio frequency (RF) thermal plasma can also spheroidise composite powders while preserving chemistry when the temperature is controlled below decomposition limits. For polyvinylidene fluoride (PVDF) with barium titanate (BaTiO_3_), a combined solid-state shear milling (S3M) and RF plasma route increased sphericity and strongly improved flow, bulk density, and powder bed quality. It also widened the sintering window and improved mechanical and functional performance in printed parts [174]. 

Beyond reshaping or surface activation, some strategies focus on engineering crystallisation and the sintering window through controlled blend-mediated thermal history, as demonstrated for a PP random copolymer in a PEO matrix.

A related blend-based strategy can widen the sintering window of commodity polymers by controlling nucleation and crystal perfection inside a sacrificial matrix. For a PP random copolymer dispersed in poly(ethylene oxide) (PEO), blend annealing increased the sintering window substantially and reduced warpage in the printed parts. This shows that thermal history engineering inside a matrix can improve process stability without changing the basic polymer family [95]. 

This section shows that powder production routes should be judged by the signatures they leave in particle size distribution tails, particle shape and roughness, surface residues, and internal porosity, because these features govern powder spreading quality, laser coupling, and build stability. It clarifies which routes tend to preserve polymer chemistry but produce angular powders, and which routes better deliver spherical particles but risk porosity or degradation. These route-to-signature links set the basis for Section 3.4, where the key powder descriptors and qualification metrics are defined.

### 3.4. Particle Attributes: Morphology, Size Distribution, and Surface Texture

The production route of polymeric powders strongly affects particle morphology, size distribution, and surface texture. Mechanical methods, like milling, tend to create angular particles with rough surfaces and broad size ranges, whereas solution-based routes more often yield spherical, smoother particles with narrower distributions [149,175]. These differences directly influence powder flow, packing, and how efficiently the laser energy is absorbed in PBF. Particle morphology governs both powder rheology and sintering. High sphericity reduces mechanical interlocking and yields smoother layers under a blade or roller. A monodisperse powder flows well but may pack with excessive porosity; controlled polydispersity fills interstices and raises packing density at the expense of cohesion if the fines content are abundant. Surface roughness increases the real contact area and capillary forces in humid air and promotes tribocharging under shear. Internal porosity, often inherited from spray or precipitation routes, lowers effective thermal conductivity and can trap gas during fusion, which influences pore formation in parts. Reporting morphology as distributions, rather than as single averages, captures tails that drive defects such as streaks, dunes, and local starvation [176]. 

In an influential morphological study on polymeric powders for PBF, the particle attributes of several engineering polymers were systematically compared, with particular attention to morphology, size distribution, and surface texture. PEEK powders that were not optimised for PBF were benchmarked against commercial PBF grades, such as PA12 and polyetherketone (PEK). Although the particle size distributions (PSD, i.e., number and volume fractions across size classes) of the PEEK grades and PBF-optimised materials were broadly similar, it was shown that PSD alone could not explain the flow behaviour. Scanning electron microscopy revealed that PA12 and PEK consisted of dense, largely spherical, or slightly elongated particles with relatively smooth surfaces, whereas non-optimised PEEK grades showed angular, flaky, fibrillated particles and mixed morphologies, including irregular plates and particles with surface protuberances. A quantitative image analysis confirmed these trends: commercial PA12-based powders exhibited the highest circularity, roundness, and solidity, and the lowest aspect ratio, while PEEK 450PF displayed the poorest values for all shape descriptors, highlighting the strong link between irregular morphology, rough texture, and poor powder performance [177]. It clearly demonstrated that, for advanced PBF feedstocks, small gains in circularity, roundness, and surface smoothness can be more decisive than modest adjustments in particle size distribution, and that targeted control of particle morphology and texture is a powerful route to engineer flowable, packable powders from otherwise unsuitable high-performance polymers [177].

The powder production route sets the particle morphology, size distribution, and surface condition, which in turn governs flowability, packing behaviour, and optical–thermal response in polymer PBF. These inherited “fingerprints” control how uniformly layers are formed and how efficiently particles fuse during laser exposure, thereby shaping density, microstructure, and defect propensity. For this reason, powder production should be treated as a primary design lever for achieving stable processing windows and repeatable part quality. 

## 4. Powder Characterisation and Qualification

Powder characterisation in this review is organised as a qualification workflow rather than as a general materials catalogue. Many laboratory tests exist, but only a subset are consistently predictive for layer formation, laser coupling, and reuse stability. This section therefore emphasises a process-relevant minimum reporting set that enables a comparison across studies and supports a root-cause analysis when builds fail. That minimum set includes particle size distributions reported with attention to fines content and distribution tails, particle morphology and surface texture as distributions rather than single averages, moisture uptake and drying sensitivity, electrostatic tendency under handling, optical response at the laser wavelength, and thermal transitions that define the usable temperature window. Where available, dynamic or temperature-relevant flow metrics are prioritised over static proxies because powder spreading is a dynamic event. This framing explains why some characterisation topics are treated briefly, while those linked directly to spreading, absorption, coalescence, and ageing are treated in depth.

### 4.1. Flowability and Rheology

Powder flow in PBF is dynamic. During spreading, the powder experiences shear, impact, and vibration that are not replicated by static tests. Nevertheless, quantitative measures guide screening. Dynamic and static avalanche angle provide rapid checks for cohesion [178,179]. Shear cell tests yield flow functions [180,181] across normal stresses relevant to spreading. Dynamic powder rheometers track the energy needed to rotate an impeller through a bed under controlled conditions and indicate sensitivity to humidity and moisture cycling [182,183]. Apparent density and tap density map the packing potential, while the spread density measured in testbeds correlates more directly with in-machine performance. Electrostatics complicate behaviour [184], humidity control [185], and temperature [29], which reduce variability. Moisture sorption increases cohesion for many polyamides and shifts sintering onset by plasticising the melt; while pre-drying and controlled storage reduce the scatter.

**Figure 9:**
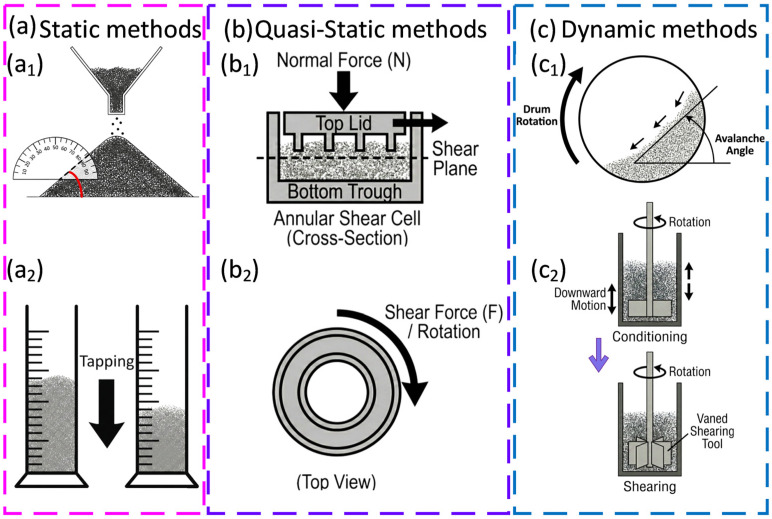
Common characterisation methods for PBF powder flow behaviour: (**a**) static methods, including (**a1**) static angle of repose and (**a2**) bulk and tap density; (**b**) quasi-static methods using (**b1**) shear testers (cross section) and (**b2**) top view; (**c**) dynamic methods, including (**c1**) dynamic angle of repose and (**c2**) powder rheometers.

Figure 9 summarises the main experimental methods used to assess PBF powder flow behaviour, from simple static tests to shear testing and dynamic rheometry.

Powder flow behaviour under realistic process temperatures has been analysed in detail using a high temperature annular shear cell, revealing how the rheology of commercial polyamide 12 (PA12) evolves as the PBF preheating conditions are approached. Cohesion, unconfined yield strength, and internal friction were measured from room temperature up to about 20 °C below the melting point, then extrapolated to zero consolidation to mimic the very low stresses experienced during powder spreading. The results show a clear optimum around 100 °C, where PA12 behaves as a free-flowing powder (flow function (ff_c > 10)), while at ambient conditions flow is hindered by moisture-induced liquid bridges and, at 140–160 °C, surface softening sharply increases cohesion and drives a transition to a cohesive or very cohesive behaviour. Importantly, the extrapolation procedure demonstrated that, in the typical processing range up to roughly 135–140 °C, the deterioration of flow is dominated by local surface deformation rather than the formation of large agglomerates, providing a quantitative basis for the preheating limits recommended for PA12 in PBF [186]. Building on this framework, the same methodology was extended to different polyamide-based powders and coupled with a granular Bond number (Bo) analysis to obtain a more universal criterion linking intrinsic flow properties to spreadability in PBF. Flow curves for PA12, an additivated PA6 grade (PA6_F) and an unmodified PA6 (PA6_0) were extrapolated to zero consolidation to estimate an isostatic tensile strength, converted via the Rumpf model into an average interparticle force, and normalised by particle weight to obtain the Bo. A clear threshold emerged: powders that produced uniform layers under spreading (PA12 and PA6_F) exhibited Bo values below about 100 in their optimal temperature window, whereas the non-spreadable PA6_0 consistently showed Bo ≫ 100, from 124 up to 485. This evolution from simple temperature-dependent shear testing to a dimensionless force-based descriptor shows that the Bo can serve as a compact, quantitative indicator of whether a given polymer powder–temperature combination will deliver stable, defect-free layers in PBF [187]. 

Powder flow behaviour for PBF can be systematically linked to layer quality by combining classical and advanced characterisation methods with direct measurements of powder bed density. In a comparative study, ten different tests were evaluated, including standardised metrics, such as bulk density (Scott volumeter), angle of repose, Hausner ratio, and discharge time, together with rotating drum measurements of dynamic angle of repose and cohesive index, and powder rheometry-based cohesion strength. Bulk density measured with the Scott volumeter proved to be the most sensitive and reliable descriptor, showing an almost perfect linear correlation with powder bed density and clearly discriminating between closely similar powder fractions, while discharge time provided a useful secondary indicator. More sophisticated dynamic methods offered additional insight into shear rate-dependent behaviour and cohesive tendencies but showed weaker or less robust correlations with layer density, and rheometry became impractical for highly cohesive, non-fluidisable powders. These results show that relatively simple bulk density measurements, when statistically correlated with powder bed density, can outperform more complex tests in predicting spreadability and packing behaviour in powder bed fusion processes, underlining that characterization techniques should be selected for their process relevance rather than their apparent sophistication [188]. 

Powder flow characterisation for PBF has been rigorously linked to final part performance by combining classical bulk tests with advanced dynamic rheology on polymeric powders. TPU and a commercial thermoplastic elastomer DuraForm^®^ Flex (DF) powders, fractionated into different particle size distributions and benchmarked against PA12, were assessed using the Hausner ratio, revolution drum analysis, and FT4 rheometry (basic flowability energy, specific energy, conditioned bulk density). Removal of fine particles systematically improved flow and packing: the coarsest, most spherical fractions showed the highest bulk packing and the lowest flow resistance, and these conditions yielded parts with higher tensile strength, larger elongation at break, increased density, and reduced porosity, directly linking close packing in the loose bed to a superior mechanical performance [189]. A complementary rheological study on blends of two commercial PA12 grades showed that dynamic FT4 metrics, such as basic flowability energy, aeration response, and conditioned bulk density, capture subtle differences in blend behaviour and correlate strongly with tensile strength, strain at break, and surface hardness of the printed parts, with a nearly linear relationship (R^2^ ≈ 0.92) between basic flowability energy and tensile strength, thereby positioning process-relevant powder rheology as a predictive tool for optimising the PBF feedstock and part quality before printing [190]. 

Comprehensive thermophysical, rheological, and optical characterisation of a commercial polypropylene powder (Ultrasint^®^ PP nat 01) has shown how powder-level metrics can be used to qualify its suitability for PBF. Morphology and particle size analysis revealed predominantly near-spherical “potato-shaped” particles with a size range of 20–80 µm, while bulk and tapped density measurements gave a Hausner ratio of about 1.23, indicating free-flowing behaviour compatible with homogeneous layer deposition. A differential scanning calorimetry and thermogravimetric analysis identified a relatively wide sintering window of 30.7 °C (with melting onset at ~133.8 °C and full fusion at around 170 °C) and high thermal stability up to ~460 °C, providing comfortable process margins for PBF without premature degradation. Hot-stage microscopy confirmed that low cooling rates and high packing density promote defect-poor fusion, whereas FTIR spectroscopy highlighted the high transmission (~98%) and low absorption at the 10.6 µm wavelength of standard CO_2_ lasers, implying that efficient processing will require the careful tuning of laser energy density rather than fundamental changes to the powder itself [94]. 

The conventional characterisation of flowability and rheology remains essential for screening polymeric powders and anticipating layer formation behaviour in PBF. However, these measurements should be interpreted as qualification indicators rather than absolute predictors, because real spreading performance is strongly shaped by machine-specific dynamics and environmental conditions.

### 4.2. Powder Spreading and Effective Parameters

In PBF, the spreading step transfers powder from the feed source to the build plate and arranges it into a uniform, mechanically stable layer suitable for subsequent fusion [191]. Previous studies have described good powder spreading as the ability of the powder to pass through the spreading tool gap to form a uniform layer only a few particle diameters thick, without empty patches, large agglomerates, or a noticeably rough surface [29,35,192]. 

The spreading tool speed determines the shear rate [36]. Higher speeds raise throughput but magnify streaks and scouring if cohesion is high. Blade or roller geometry sets contact length and pressure distribution [193]. The spreading tool geometry strongly governs how powder is mobilised, compacted, and finally deposited into a stable layer in PBF. Rounded geometries (e.g., roller and round-edge blades) promote smoother engagement at the pile front, enabling better particle rearrangement and stronger, more continuous force transmission into the bed. As a result, they tend to produce higher packing fraction and spreading density ratio, with lower surface roughness—i.e., denser and flatter layers. By contrast, sharp and flat blades intensify shearing and accelerate particles, which delays settling and increases voids and surface irregularity, especially as the spreading speed rises. The geometry–speed interaction determines whether the powder layer forms as a uniform, well-compacted layer or as a rough, underfilled layer that can amplify downstream defects and variability [194]. A flexible spreading tool can accommodate large particles but may leave ridges; a rigid spreading tool levels well but is sensitive to agglomerates [195,196]. The effective layer thickness, that is the gap between the spreading tool and the build plate, should be larger than the coarsest particles in the powder (typically about 2–3 d_90_) to minimise layer gouging and powder jamming [197]. The bed temperature couples to the spreading because soft particles deform, which can degrade packing or exacerbate aggregation depending on the surface energy [25,30,198]. 

**Figure 10:**
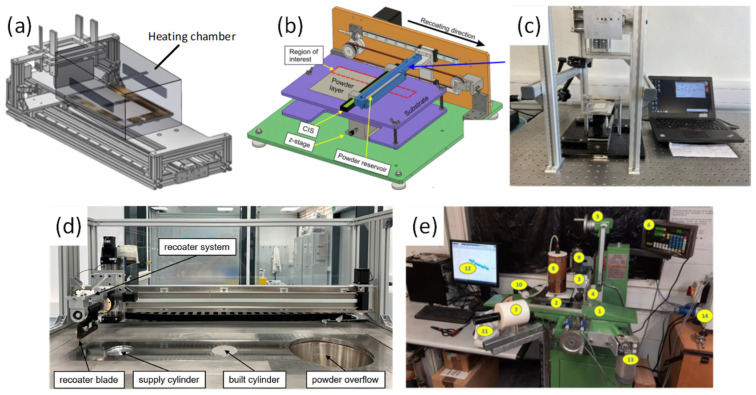
Examples of custom-built powder spreading devices developed for ex situ studies of PBF powder spreading behaviour: (**a**) spreading device with integrated bed heating (reproduced from [25] Elsevier, 2024); (**b**) device enabling different spreading strategies (reproduced from [199], Elsevier, 2021); (**c**) rig with a single-bed pocket configuration (reproduced from [24], Elsevier, 2023); (**d**) spreading device equipped with a digital microscope (reproduced from [200], MDPI, 2025); (**e**) modified industrial machinery adapted for powder spreading experiments (reproduced from [201], Elsevier, 2022).

Powder flow behaviour for polymer PBF has been increasingly quantified using process-relevant spreading tests rather than only classical bench rheology. Figure 10 shows several custom-built powder spreading devices designed to study PBF powder spreading behaviour under controlled, ex situ conditions. These rigs enable systematic variation of process parameters such as bed heating, spreading strategy, and in situ imaging to better understand layer formation mechanisms. 

Powder flow and layer formation for PBF have been examined using increasingly process-relevant imaging and spreading methodologies that focus on polymer powders such as PA12, polystyrene (PS), polymethylmethacrylate (PMMA), PP, PA6, and TPU. A lab-scale blade spreading tool derived from a film applicator was used to mimic PBF spreading step and to define a packing ratio, obtained as the ratio between layer density and tapped density, which isolates the effect of flowability on layer quality. Spherical PA12, PS, and PMMA powders produced smooth, defect-free layers with packing ratios typically above 0.9, whereas cryogenically milled TPU showed severe defects or even incomplete layers despite apparently acceptable packing, highlighting the influence of elasticity and compressibility on true spreadability. A complementary spreadability tester based on a precision linear stage and a shadowgraphy set-up quantified powder bed roughness through grey-scale amplitude and roughness wavelength, revealing that cohesive polymer powders develop higher surface roughness and characteristic groove defects than free-flowing grades, and that the spreading tool geometry and nominal gap size, expressed relative to particle d_90_, strongly modulate these effects [201,202]. 

Microscopic and macroscopic image analyses have further deepened understanding of polymer layer formation by linking optical roughness descriptors directly to processing conditions. A wavelet analysis of grey-level profiles from PA6, PA6 black, PP powders, and TPU was used to define a characteristic roughness length scale, normalised by median particle size, showing that slower spreading speeds can improve layer homogeneity for certain materials, but that spreadability rankings do not necessarily follow conventional flow indices, such as the Jenike flow factor. A heated spreading rig then demonstrated that layer quality can deteriorate markedly with temperature even when classical flow tests still classify the powders as free-flowing: TPU loses spreadability near 110 °C, PP powders and PA6 black grades show progressive loss of coverage and increased uncovered fraction, whereas PA6 maintains an acceptable layer quality up to the same temperature. These findings emphasise that polymer spreadability in PBF is governed by a coupled response of particle shape, viscoelasticity, and interfacial behaviour with the underlying bed, and must therefore be characterised with dedicated, process-mimicking tests rather than room temperature flow metrics alone [25,35].

Optimised conditions are those that produce uniform surface height and stable apparent density, while preserving particle integrity. In a study using milled polyaryletherketone (PAEK) powders designed for PBF, powder rheology and particle size–shape analyses were combined to link flow behaviour directly to spreading performance under processing conditions. Basic flow energy (BFE) was shown to increase with conditioned bulk density (CBD) and to decrease with compressibility (CPS), indicating that a higher flow energy is associated with dense but less compressible beds, particularly when particles are more spherical, as reflected by a strong positive correlation between circularity and CBD and a negative correlation with CPS. A key outcome was that the traditional focus on median size (d_50_) is insufficient for flow design, since the fine fraction (d_10_) correlated strongly with permeability (PD) and the flow rate index (FRI), with a higher content of fines promoting dense, low-permeability beds that resist gas flow. Spreading trials at an elevated temperature on PEEK, PEK, and PEEK–PEDEK (PDK) grades showed that successful, defect-free layers are best predicted not by single bulk indices but by normalised aeration sensitivity (NAS), with powders exhibiting an NAS > 0.31 s/mm consistently passing spreading without agglomeration. Thermal conditioning improved CBD and, when combined with favourable NAS and permeability, promoted more reliable layer formation, underlining that optimal spreading of high-performance PAEK powders in PBF requires simultaneous control of particle shape, fines content, and aeration response rather than particle size alone [203]. 

Powder spreading performance and its governing parameters largely determine powder bed uniformity, which directly controls fusion stability and defect sensitivity in polymer PBF. For reliable qualification, spreading should be evaluated through both measurable powder descriptors and layer-level metrics that reflect the real powder spreading conditions.

## 5. Process Parameters and Strategies

Because polymer PBF exhibits narrow and powder-dependent stability windows, the goal of this section is to provide process maps and dependencies, not a single universal parameter set. Readers are guided to (i) select layer thickness consistent with the powder’s upper size tail, (ii) stabilise the thermal field via preheating close to the onset of melting without triggering sintering during spreading, and (iii) tune the laser power/scan speed/hatch spacing together while monitoring defects associated with under- and overexposure.

### Energy Input, Scan Strategy, and Thermal Management

Laser power, scan speed, hatch spacing, and exposure strategy define the energy density delivered to the powder bed [204]. The goal is to reach a melt fraction that forms stable necks without causing excessive melt pool growth or stair-step rounding [205]. Energy density alone is an incomplete descriptor because optical absorption depends on pigment content [206], particle packing [207], and the local surface [208]. Preheating the bed close to the onset of melt reduces the additional energy needed [209] but, if the temperature is too high, the powder may sinter during spreading and degrade during long dwell times. Scan strategy controls heat distribution and accumulation [210]. Alternating hatch directions, dividing large areas into islands, and optimising contour timing can manage residual stress and reduce curl [79]. Layer wait time and gas flow rate adjust convective losses and smoke removal. Atmosphere composition limits oxidation; for oxygen-sensitive polymers, low oxygen partial pressure is essential during long builds [211]. 

Key process parameters, such as laser power (*P*), scan speed (*v*), hatch spacing (*h*), and layer thickness (*t*) together control how much energy is delivered to the powder and, consequently, the degree of melting and bonding. Their combined effect is often expressed through the volumetric energy density (*E_V_*), as given in Equation (1):

(1) $$E_{V}=\frac{P}{v.h.t}$$

Control of process parameters in PBF for polymeric powders has been shown to be critical for balancing defect-free fusion with avoidance of thermal damage, particularly when processing semi-crystalline polymers, such as high-density polyethylene (HDPE). By decoupling bed temperature from laser energy density, it was demonstrated that raising the powder bed to 125 °C stabilises the thermal field and largely suppresses curling, while tuning the linear energy density in the range of about 4.5–9.1 J/m leads to a clear optimum around 7.4 J/m, where nearly full densification (≈1.7% porosity) and an ultimate tensile strength of about 26 MPa are achieved. Beyond this optimum, further increases in input energy cause over-melting, local degradation, and irregular void formation, which reduce mechanical performance despite higher nominal energy input, illustrating that energy density must be optimised rather than simply maximised [93]. Extending this process-centric view to medical-grade PA12, systematic variation of laser power, scan speed, and layer thickness has clarified how input energy and its distribution in space and time govern both microstructure and functional performance for biomedical components. Moderate laser power (30 W), intermediate scan speed (≈750 mm/s), and a 100 µm layer thickness were identified as an optimal window, providing continuous melt tracks, low surface roughness (≈2.5–3.8 µm), and a favourable crystalline structure that supports high strength and wear resistance, whereas lower energy inputs produced porous, incompletely fused surfaces and higher powers (40 W) induced micro-cracks and burnt regions linked to thermal degradation. These results emphasise that suitable PBF process windows for polymers are defined by a narrow balance between sufficient energy for crystallinity and coalescence and an upper threshold beyond which part integrity and long-term stability are compromised [212]. 

Process optimisation for PBF of phenolic thermoset (PF) composites modified with PA12 has been shown to depend strongly on laser input energy and the resulting energy density, defined as laser power divided by the product of hatch spacing and scan speed. By systematically varying power and scan speed for different PA12/PF ratios and analysing porosity via X-ray computed tomography, an optimal formulation of 30/70 PA12/PF was identified, with a laser power of 8 W and a scan speed of 250 mm/s, corresponding to an energy density of about 0.213 J/mm^2^ within an effective processing window of 0.2–0.3 J/mm^2^. Energy densities below this range led to incomplete fusion and weak crosslinking, whereas higher values caused thermal degradation, increased porosity, and poor interlayer bonding, underlining that both under- and overexposure are detrimental to structural integrity. Subsequent multi-stage thermal post-curing further reduced porosity by roughly 8–10% and increased flexural strength by about 14–16%, confirming that careful tuning of laser energy input, in combination with controlled post-processing, is essential for qualifying thermoset–thermoplastic composite powders for high-performance PBF applications [47].

The influence of building strategy, build orientation, and hatch orientation on the performance of PA12 parts has been clarified by comparing different PBF routes and scanning strategies. Using HP Multi Jet Fusion (MJF) and PBF, anisotropic behaviour was mapped over nine orientations, showing that mechanical properties cannot be described only with respect to principal axes: rotations within the X–Y plane and along the Z plane significantly modified tensile strength and porosity, with MJF parts in the Z plane orientation reaching roughly 25% higher strength than the other orientations and PBF counterparts, while PBF remained more sensitive to orientation but often delivered higher modulus and elongation. In a study of PBF using PA12 on industrial equipment, variations in laser power and hatch direction further demonstrated that mechanical response is tightly coupled to how energy is distributed in the powder bed: increased laser power improved coalescence and reduced “cauliflower-like” unmelted particles, and a classical 0°/90° alternating hatch pattern became effectively orientation-independent at high power; whereas, at lower power, the 45° orientation showed inferior tensile strength, while post-build annealing at 170 °C for several hours relaxed residual stresses and increased crystallinity, further enhancing stiffness and strength [213,214]. A more detailed view of the geometrical side of the process strategy was provided by systematically varying the build orientation and wall thickness for PA12 parts while keeping the hatch pattern fixed, revealing that sample thickness could influence anisotropy more strongly than orientation itself. Thin PBF walls (0.8 mm) were found to be around 20% less stiff and 27% weaker than thicker ones (3.2 mm), due to a larger proportion of edge regions suffering from incomplete sintering, while vertically built specimens (Z plane oriented) exhibited the poorest yield and ultimate strength because of higher porosity and weaker interlayer bonding; by contrast, Poisson’s ratio remained nearly insensitive to orientation under a constant hatch layout. Together, these studies show a clear evolution from simply recognising anisotropy, to tuning laser power and hatch orientation to mitigate it, and finally to integrating build orientation and thickness as designable parameters in the process window for high-performance PA12 PBF components [215].

Cooling time was exploited as a key process parameter to tune crystallisation and mechanical response of high temperature PEKK parts produced by PBF, showing that slow, furnace-like cooling leads to highly crystalline, strong, but very brittle components with elongation at break below about 2.5%; whereas, interrupting cooling and quenching the build after shorter dwell times produces low-crystallinity structures with a dramatic increase in ductility, reaching roughly 14% elongation after 1 h of cooling for only a modest loss in tensile strength. A sharp transition in crystallinity was identified between about 6 h and 7 h of cooling, with an intermediate crystallinity of ~16% giving the best compromise between stiffness and toughness, with very short cooling times even promoting the formation of a distinct PEKK crystal form II. These relationships were condensed into a calibration curve linking cooling history, crystallinity, and mechanical performance, highlighting that active control of post-build cooling in PBF can be used as a powerful lever to overcome the traditionally brittle behaviour of PAEK powders and to tailor PEKK parts for different application targets [98]. 

**Figure 11:**
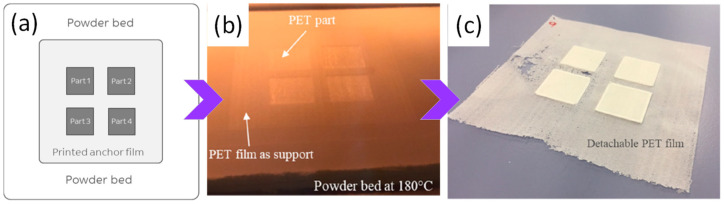
Anchor film approach for low-temperature PET printing: (**a**) print layout; (**b**) in situ printing of four PET plates on an ~70 μm anchor film at a reduced bed temperature of 180 °C; (**c**) PET plates after removal from the build bed with the anchor film still attached. Reproduced from [216], MDPI, 2024.

Temperature is treated as a central design variable rather than as a simple setting in PBF, showing how careful control of the powder bed history can either engineer or deliberately suppress crystallisation during the process. For high temperature PEEK, detailed isothermal and non-isothermal crystallisation kinetics revealed that a theoretical crystallisation limit at about 321 °C must be shifted to a practical bed temperature near 332 °C to counteract cooling from a cold powder coating (CPC), with a maximum crystallisation rate around 250 °C identified as the minimum powder feed temperature; maintaining a high, quasi-static bed temperature (≈330 °C) was shown to promote larger, better oriented crystallites and high mechanical strength, while shrinkage was linked specifically to the subsequent dynamic cooling stage [99]. Building on this thermally informed view of process control, a complementary strategy was proposed for high-melting polymers, such as polyethylene terephthalate (PET) and PA12, where an in situ printed, flexible anchor film (see Figure 11) enabled stable PBF at bed temperatures far below the usual processing window (down to ~150 °C for PET and ~140–150 °C for PA12) with only modest density losses at 170–200 °C and acceptable consolidation at reduced temperatures, thereby extending material compatibility, reducing degradation and energy demand, and demonstrating that temperature can be “traded” against mechanical restraint to keep warpage and curl under control in polymer powder bed fusion [216]. 

Overall, energy input, scan strategy, and thermal management jointly define the thermal history in polymer PBF and therefore control interlayer bonding, crystallisation behaviour, and residual stress development. When these parameters are balanced within a stable process window, dense parts with consistent microstructure and dimensional accuracy can be produced. Conversely, local over- or underexposure and poor heat management readily translate into porosity, warpage, and other process-induced defects

## 6. Microstructure, Defects, and Process Windows

The thermal history through each layer sets the crystalline morphology and residual stress of the part. The cooling rate determines the spherulite size and the crystalline fraction. Slowly cooled regions develop higher crystallinity and stiffness but also larger shrinkage, which can drive distortion adjacent to thin walls and sharp corners. Reheating from subsequent layers anneals prior materials and can relax stress or coarsen crystallites. The interplay between contour and hatch influences local densification and surface finish. Amorphous systems show different pathways: densification proceeds through viscous flow, and glass transition kinetics dominate shape retention. For both classes, porosity arises from trapped gas, insufficient neck growth, or lack of fusion; these defects degrade strength and fatigue resistance. Thermal gradients at overhangs and near large voids require careful energy management to avoid sag and closed porosity. 

Defects in polymer PBF can be organised into four mechanism-based groups: (i) Powder-bed defects (streaks, voided tracks, unfilled areas, roughness bands, local density gradients) originating from spreading instability, cohesion, tribocharging, and temperature-softened particle interactions; (ii) Fusion defects (lack of fusion, incomplete neck growth, interlayer weakness, trapped unmelted particles) driven by insufficient or non-uniform energy delivery and by local packing variations; (iii) Thermomechanical defects (curling/warpage, delamination, residual stress cracking) governed by thermal gradients, crystallisation shrinkage, and scan strategy–induced heat accumulation; and (iv) Thermochemical defects (discoloration, embrittlement, microcracks, degraded surfaces) caused by overheating, oxidation, and ageing during long builds and repeated reuse.

**Figure 12:**
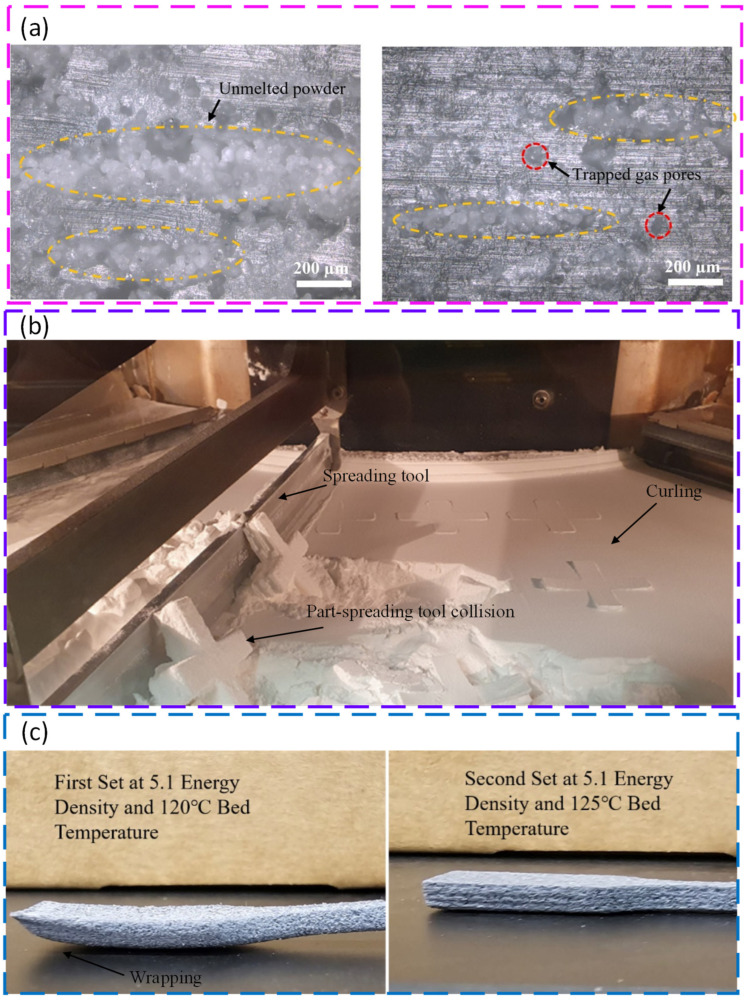
Representative defects in PBF of polymeric powders: (**a**) lack of fusion with unmelted particles and gas entrapment in PA12 (reproduced from [217], Virtual and Physical Prototyping, 2023); (**b**) curling and process interruption due to collision between the printed part and the spreading tool in PA12 (reproduced from [224], Elsevier, 2022); (**c**) warping defects in HDPE observed under different process parameters (reproduced from [93], Elsevier, 2025).

Common defects include lack of fusion (unmelted particles) [217], porosities [218], over-melting with edge rounding [219], balling of the melt track [220,221], stair-step roughness [222,223], curl [224], and warpage [93]. Lack of fusion occurs when local energy is inadequate or when voids in the powder layer reduce heat coupling. Over-melting can coalesce pores and erode sharp features. Balling results from poor wetting and Rayleigh instabilities in thin tracks and is more severe with low-viscosity melts on cool beds. Curl and warpage follow from uneven shrinkage during crystallisation and from constrained contraction near rigid features. A practical process window expresses bounds on layer temperature, energy input, and scan strategy that avoid these defects for a given powder and geometry. Mapping this window demands both in situ observation and destructive evaluation because visual surface quality may not reveal subsurface lack of fusion. Figure 12 illustrates typical defects in polymer PBF parts, including lack of fusion and gas entrapment, curling leading to build interruption, and warping induced by unsuitable process parameters. 

**Figure 13:**
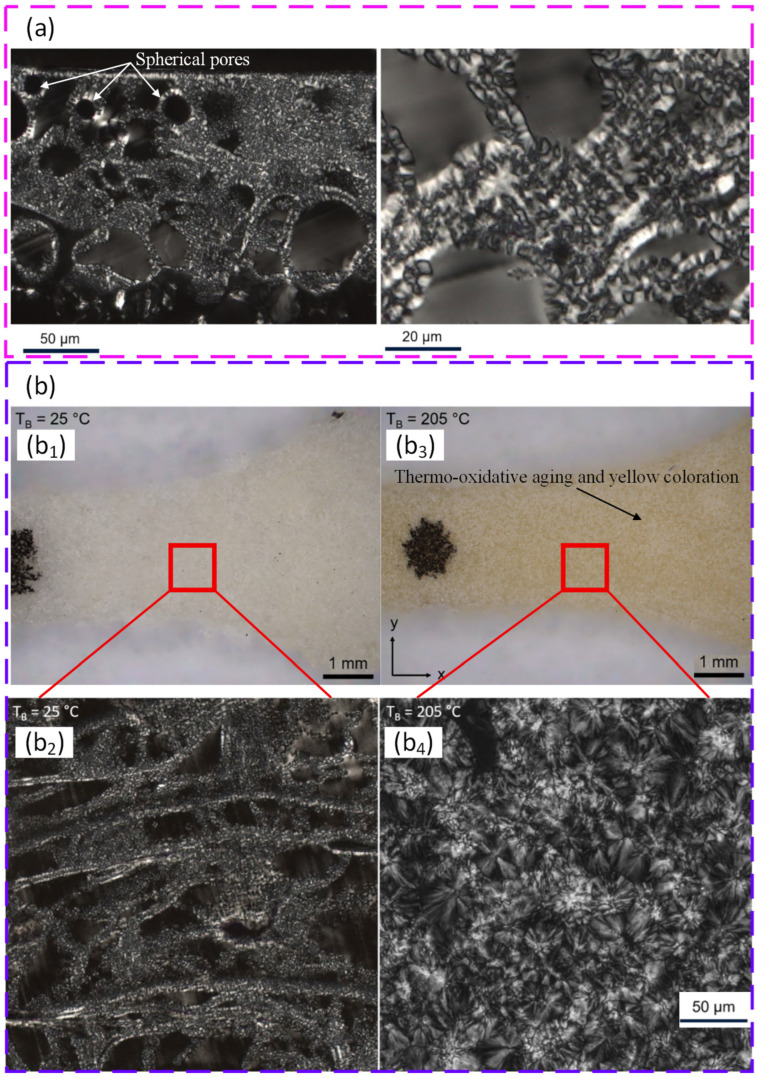
Microstructural evolution and oxidation of PA6 under non-isothermal and quasi-isothermal PBF: (**a**) single-layer microstructure at 25 °C showing spherical pores (two magnifications); (**b**) multilayer parts, including (**b1**,**b2**) appearance and microstructure at 25 °C (non-isothermal; dense with microspherulitic regions) and (**b3**,**b4**) appearance and microstructure at 205 °C (quasi-isothermal; yellowing and large spherulites). Reproduced from [87], Springer Nature, 2025.

Non-isothermal PBF has been shown to expand the usable process window for PA6 by shifting the focus from a narrow quasi-isothermal plateau to a time-resolved thermal control. Stable consolidation was achieved at bed temperatures as low as 25–75 °C using cryo-milled PA6 and a locally quasi-simultaneous exposure strategy, which generated very high cooling rates (>−250 °C/s). Under these transient conditions, large spherulites were suppressed and the fraction of the stiff α-phase was reduced in favour of finer microspherulitic morphologies. This approach also reduced thermo-oxidative degradation, as suggested by the absence of yellowing and FTIR evidence of chain scission. A key practical insight is that multilayer density can remain high even when single layers show spherical pores and incomplete local coalescence, because repeated exposures can act as in situ annealing and complete cold crystallisation of metastable material. The same mapping also clarifies the new limits: very low bed temperatures can improve layer homogeneity, while higher “non-isothermal” temperatures can increase intralayer curling and surface topography defects. In other words, the PA6 process window becomes strongly dependent on exposure discretisation and thermal transients, not only on bed temperature relative to melting and crystallisation [87]. Figure 13 shows how non-isothermal versus quasi-isothermal PBF conditions modify PA6, from more porous, nearly colourless structures at low bed temperatures to dense, yellowed microstructures with coarse spherulites at high bed temperatures. 

**Figure 14:**
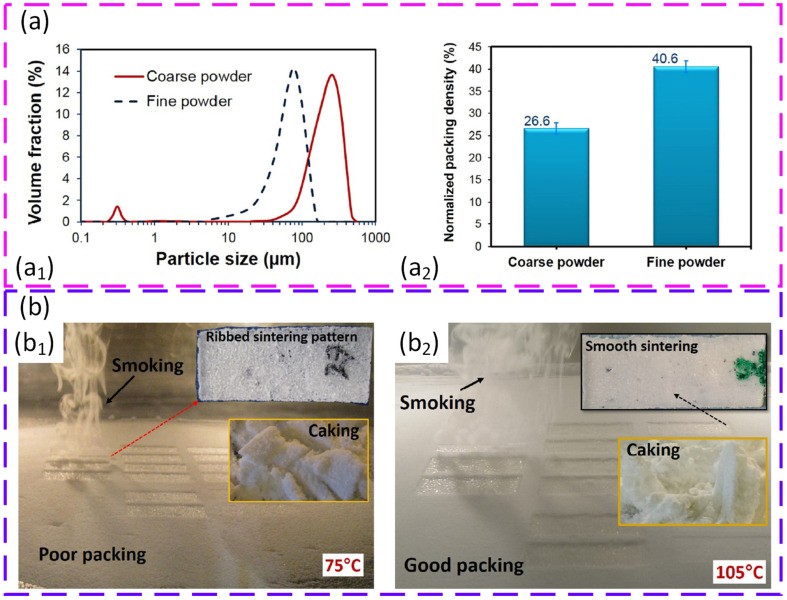
Effect of particle size and packing density on the quality of 3D-printed TPU layers by PBF: (**a**) powder properties, including (**a1**) particle size distribution and (**a2**) packing density of fine and coarse powders; (**b**) PBF process and associated visual defects, highlighting (**b1**) coarse powder with poor packing and a ribbed printing pattern, and (**b2**) fine powder with good packing and a smooth printed surface. Reproduced from [21], Elsevier. 2016.

A similar “window sensitivity” appears for thermoplastic TPU, where defect formation follows a direct chain from powder packing to melt coalescence. Fine TPU powder processed at an optimised bed temperature of 105 °C can pack efficiently and sinter smoothly, enabling relative densities near 97% and tensile response close to the injection-moulded references. By contrast, coarser powders and lower bed temperatures (for example 75 °C) reduce packing quality, increase internal porosity, and weaken coalescence. The broader trend is that, for elastomers, small shifts in bed temperature and particle size distribution can move the process from stable densification to persistent porosity and weak bonding, even when other settings remain unchanged (see Figure 14) [21]. 

**Figure 15:**
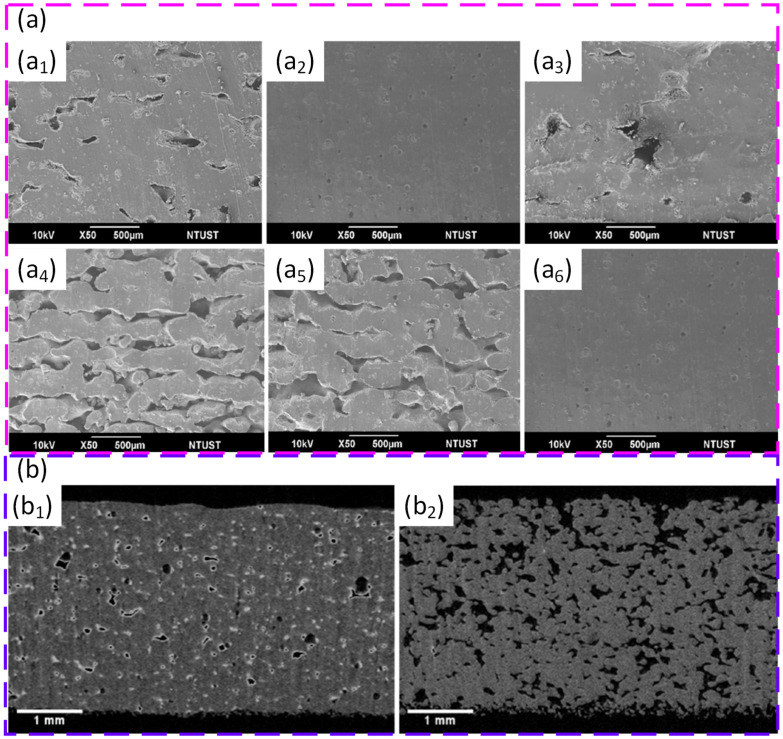
Microstructural evolution and defect distribution in carbon black-modified TPU: (**a**) interlayer adhesion at different layer thicknesses (**a1**) 100 μm, (**a2**) 125 μm, (**a3**) 150 μm, and effect of preheating at a constant laser energy density of 0.028 J/mm^2^ with bed temperatures of (**a4**) 35 °C, (**a5**) 55 °C, and (**a6**) 75 °C; (**b**) micro-CT porosity maps of (**b1**) carbon-black-modified TPU and (**b2**) commercial TPU. Reproduced from [125], MDPI, 2024.

Once powder packing becomes adequate, laser–powder coupling often becomes the next dominant lever, especially for optically reflective polymers. Carbon black-modified TPU demonstrates how surface chemistry can be used to tune absorption and to reduce defect populations, but only inside a narrow additive–process balance. A thin, uniform carbon black coating applied at 70 °C can smooth the particle surface and reduce reflectivity, promoting deeper energy absorption and more complete melt coalescence. By contrast, room temperature mixing or excessive heating can produce poorly bonded coatings or local fusion of grains, which undermines the feedstock quality. Under optimised conditions (preheating 75 °C, energy density 0.028 J mm^−2^, layer thickness 125 µm), the resulting parts show large continuous melt regions, fine microporosity, and no obvious interlayer gaps, while non-optimal energy input or layer thickness preserves a grain-resolved microstructure with interconnected pores. Micro-CT confirms that these settings suppress voids and interface defects compared with commercial TPU powders. Importantly, higher carbon black content can again degrade fusion by reducing the effective polymer–polymer contact area. The practical message is that optical absorbers should be treated as process enablers with an optimum, not as monotonic “more is better” additions (see Figure 15) [125]. 

Shrinkage-driven distortion further shows why defect mitigation cannot be reduced to energy density alone. A comparative analysis of semi-crystalline PEKK and PA12 decomposed volumetric shrinkage into thermal contraction, crystallisation shrinkage, and powder-related contributions. In PA12, crystallisation accounted for roughly 60% of the total shrinkage, making crystallisation shrinkage a dominant driver of warpage and curling when the process window is not centred appropriately between melting and crystallisation. In PEKK, irregular and porous powder morphology increased the powder-related shrinkage contributions to about 57–58%, yet overall shrinkage was about 30% lower than for PA12. For small parts (<10 mm height), net expansion could even occur due to upskin/downskin effects from through-layer energy transmission. This comparison highlights a useful rule for high-performance polymers: process windows must be defined using both thermal transitions and the mesostructure inherited from the feedstock, because powder morphology can shift distortion behaviour even when the polymer family is “advanced” [100].

Mechanistic modelling has also clarified why some defects remain stubborn even when surfaces appear acceptable. A coupled DEM–CFD framework resolved powder spreading, melt flow, and pore evolution in PA12 and showed that very fine particles (<55 µm) can increase bed cohesion, degrade powder spreading, and promote lack-of-fusion porosity. At the same time, gas entrapment during coalescence can generate nearly spherical pores even under otherwise favourable conditions. By varying laser power, a narrow but practical window was identified in which porosity dropped from 33.7% at 7.2 W to 7.5% at 14 W, with an optimised regime near 3.5% porosity when viscosity and energy input jointly supported air evacuation without over-melting. A key trend emerges: acceptable microstructures typically sit in a narrow energy band where lack-of-fusion and gas entrapment mechanisms are controlled at the same time, which explains why “good-looking” surfaces can still hide internal porosity [217].

**Figure 16:**
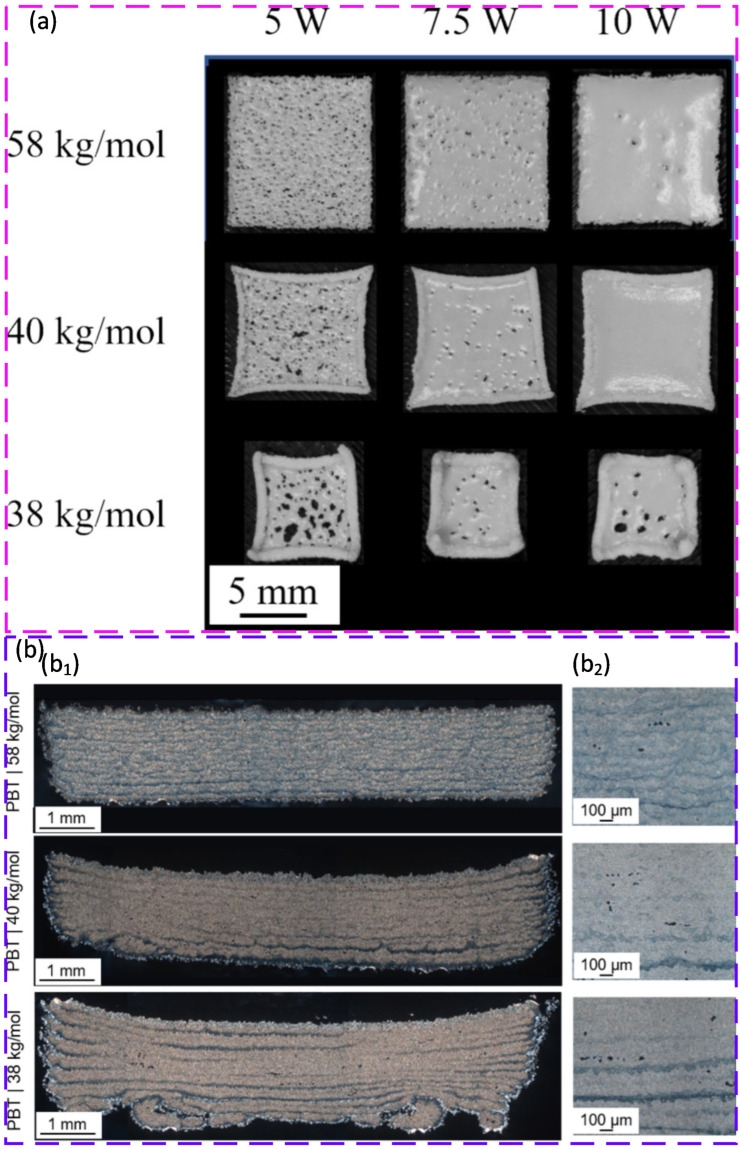
PBT powders in PBF: (**a**) influence of molar mass and laser power (5, 7.5, 10 W at 2000 mm/s and 0.2 mm hatch) on contraction and distortion of single layers; (**b**) effect of increasing molar mass on (**b1**) macroscopic part quality (balling/contraction at 38–40 kg/mol versus sharper edges at 58 kg/mol) and (**b2**) cross-sections (dense fusion for all; unmelted particles mainly near surfaces; pores < 50 µm for 58 kg/mol). Reproduced from [91], Springer Nature, 2025.

PBT offers a clean example of how melt rheology can dominate defect formation even when powder morphology is favourable. Precipitated PBT powders with low, medium, and high molar mass can share similar spherical particles, fine spherulitic microstructures, and low internal porosity (≈0.36–1.39%), yet behave very differently under laser exposure. High viscosity PBT can provide a wider and more robust process window, with geometrically accurate parts, flat surfaces, sharp edges, high fusion density, and only minor distortion. Low-viscosity PBT can undergo rapid melt-pool contraction (“balling”), causing local geometry losses exceeding 50% and making stable horizontal layers difficult to maintain. Since surface tension was similar across grades, the instability was attributed to viscosity falling below a critical threshold (about 400 Pa·s), where surface tension dominates viscous resistance. This case reinforces a practical interpretation: when viscosity is too low, defect control becomes difficult even if packing and densification appear favourable. At the same time, brittle fracture remained common across grades, showing that removing porosity and macro-defects does not automatically overcome the intrinsic brittleness linked to a crystalline microstructure [91]. Figure 16 shows how PBT molar mass and laser power affect single-layer distortion and part quality, with a higher molar mass yielding sharper edges, fewer defects, and dense microstructures. 

The microstructure and defect landscape in polymer PBF is set by coupled constraints rather than by single “optimal” settings. The most reliable process windows are those that stabilise powder spreading, maintain consistent laser–powder coupling, control crystallisation shrinkage, and limit thermochemical drift during long builds. For many polymers, defects shift abruptly once these couplings drift outside a narrow band, which supports using process maps and targeted validation builds to confirm stability. Defect mitigation therefore benefits from treating powder descriptors, thermal management, and melt kinetics as one system, which is also the most direct path toward engineering new polymer–process combinations around defined performance targets.

Table 1 provides a concise checklist of key powder descriptors and typical process window ranges for common PBF polymers to support comparison and stable builds. The main message is that parameters must be treated as a coupled set: layer thickness should be selected relative to the PSD, and hatch spacing, scan speed, and laser power should be balanced together because each change shifts the practical energy input and melt behaviour. The table also highlights the growing importance of atmosphere control for high temperature polymers, such as PEEK, and the need to keep the bed or chamber temperature close to the relevant thermal transition region to limit warpage while preserving a stable sintering window. The reported ranges are intended as guidance rather than as fixed settings because they depend on machine architecture, optics, powder spreading strategy, powder history, and part geometry. Reuse and refresh are included as boundary conditions and should be reported explicitly, with further discussion provided in Section 7.

## 7. Sustainability and Lifecycle

### Powder Ageing, Reuse, and Refresh Practice

From an economic perspective in AM, PBF strongly encourages powder reuse [233,234]. Reuse reduces cost and waste, but it also introduces a predictable technical risk: repeated exposure to elevated temperature and oxygen gradually shifts the powder state and narrows process stability. The dominant mechanisms are well known. Chain scission lowers molecular weight and viscosity; while crosslinking or post-condensation increases viscosity and slows coalescence. Oxidation shifts colour and produce carbonyl signatures [87]. Thermal history can broaden molecular weight distribution and alter crystallisation kinetics. These effects do not simply accumulate in a linear way. They depend on polymer chemistry, stabilisers, build temperature, residence time, humidity history, and oxygen ingress. A practical implication is that reuse should not be treated as a fixed number of cycles. A more reliable approach is to treat powder as a monitored consumable whose state is tracked and managed. Refresh strategies, typically by blending used powder with virgin powder, help stabilise properties across builds. The optimal refresh ratio is therefore polymer- and machine-dependent, and it should be selected based on measurable drift rather than habit. Monitoring methods that are routinely available in laboratories and production settings are sufficient for early warning. Melt flow index or rheometry can capture viscosity drift. DSC peak positions and enthalpies provide evidence of changes in melting and crystallisation behaviour. Infrared spectroscopy provides rapid indicators of oxidation. Moisture control remains essential because ageing can change surface chemistry and moisture sorption behaviour, which then affects cohesion, electrostatics, and powder spreading stability. Handling protocols that reduce residence time at temperature and limit oxygen ingress are therefore directly linked to sustainability because they extend usable powder life while reducing variability.

The reuse behaviour of PEEK illustrates why “sustainability” in PBF is not only a materials issue but is an atmosphere and thermal history issue. Thermal exposure of PEEK 450PF powder at 330–340 °C for up to 12 h showed that the powder can be chemically robust when protected from oxidation, with negligible degradation signatures in FTIR and TGA and little change in particle size distribution and morphology [228]. The most significant ageing effects were microstructural and rheological. Crystallinity and crystal size increased rapidly early in exposure and then reached a plateau, while melt viscosity and elasticity increased markedly. This behaviour is consistent with molecular weight growth or post-condensation and implies slower sintering kinetics for reused powder. Flowability remained in the “poor” regime but could show slight improvement after annealing, meaning that powder spreading can remain feasible, yet process parameters and refresh practice must account for slower coalescence kinetics [229].

**Figure 17:**
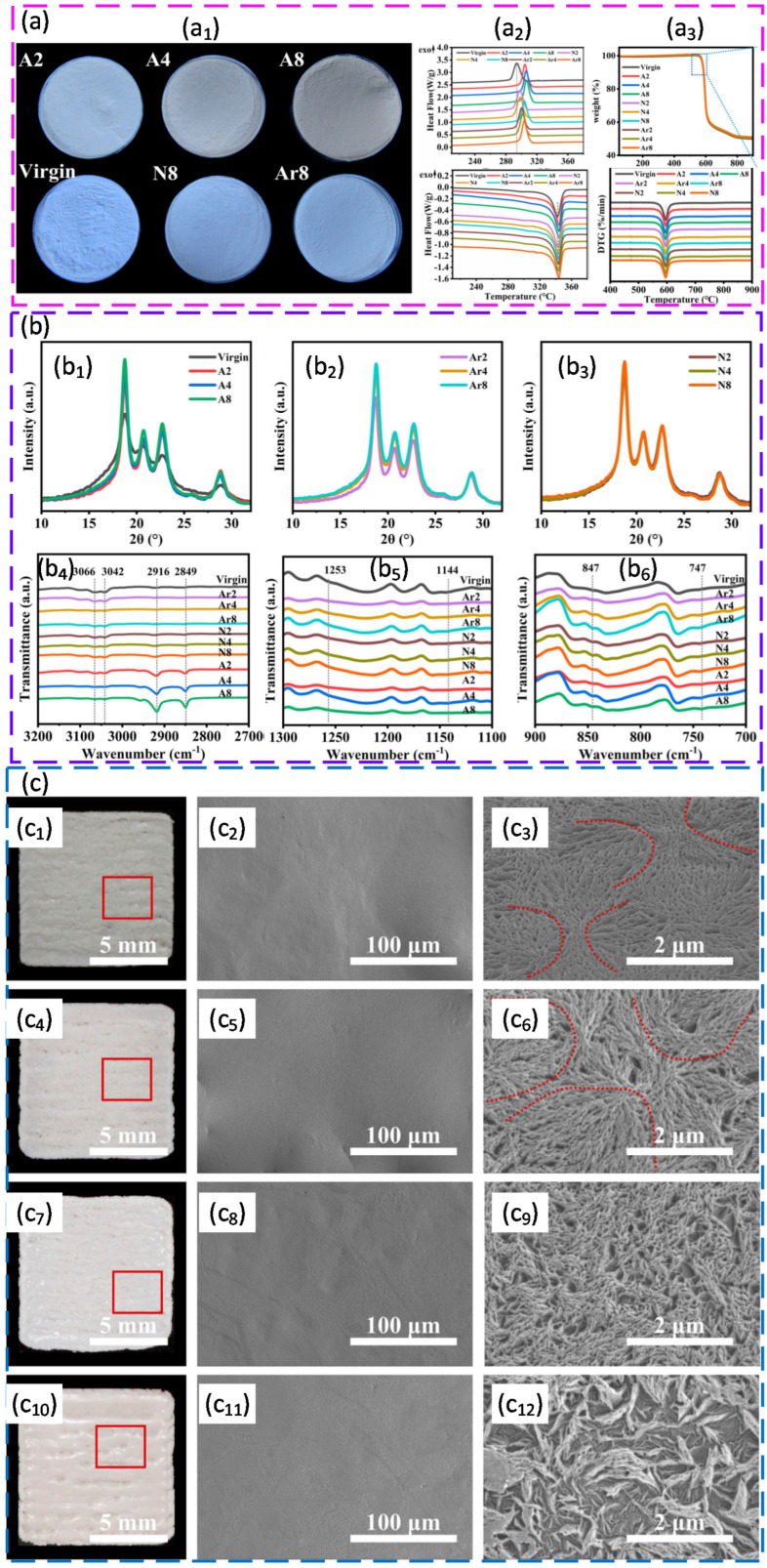
Thermal ageing of PEEK powders at 330 °C in air, argon, and nitrogen: (**a**) powder characterisation including (**a1**) appearance, (**a2**) DSC, and (**a3**) TGA/DTG; (**b**) XRD in (**b1**) air, (**b2**) argon, and (**b3**) nitrogen, and FTIR in (**b4**) 3200–2700, (**b5**) 1300–1100, and (**b6**) 900–700 cm^−1^; (**c**) single layers and SEM for virgin powders in (**c1**–**c3**) argon and (**c4**–**c6**) nitrogen, and aged powders (Ar8, N8) in (**c7**–**c9**) argon and (**c10**–**c12**) nitrogen (arcs mark crystalline boundaries). Reproduced from [229], Taylor & Francis. 2023.

Atmosphere effects further clarify the boundary between reusable and unusable powder. When PEEK powders were held at 330 °C under air, nitrogen, and argon, the atmosphere strongly controlled the degradation pathways and practical reprocessability (see Figure 17) [229]. In inert gases, crosslinking and changes in crystallisation behaviour narrowed the sintering window and shifted the energy balance. Exposure in air caused severe oxidative degradation, rendering the powder unusable. Aged powders under nitrogen and especially argon remained structurally usable but required adaptation of energy input to avoid over-melting and surface damage. A conservative interpretation is that a “recyclable loop” for high temperature polymers is realistic only when atmosphere control is treated as a primary sustainability lever rather than as a secondary setting [229]. 

For commodity polymers, reuse tolerance can appear high in datasheets yet remain limited in precision practice. A dedicated polypropylene grade (Laser PP CP 75) showed moderate melt flow changes across reuse cycles and a progressive increase in the melting point and sintering window, indicating a drift toward higher required energy input with accumulated thermal history [96]. The surface quality remained free of the “orange peel” effect typical for PA12 [235], but geometric fidelity deteriorated after several cycles. Edge distortion and increasing dimensional error eventually caused build interruption, so an unlimited closed-loop reuse was not achieved for high-precision builds [96]. This highlights a key trend: the sustainability limit is often set by dimensional drift and defect probability, not by whether powder can still fuse. A similar pattern emerges for bio-based PA11, where full reuse without refreshing remained feasible only for a limited number of cycles before ageing-driven embrittlement and defect formation dominated [85]. Early reuse could improve utilisation and part density, consistent with modest molecular weight growth. With further cycling, high viscosity inclusions and unmelted regions increased, surfaces became coarser, brittleness increased, and shrinkage and dimensional drift became more pronounced. These observations support a broad recommendation. Sustainable practice requires reuse and refresh strategies tuned to measurable evolution in rheology, crystallinity, and the process window so that material savings do not silently trade off against mechanical reliability or dimensional accuracy [85].

**Figure 18:**
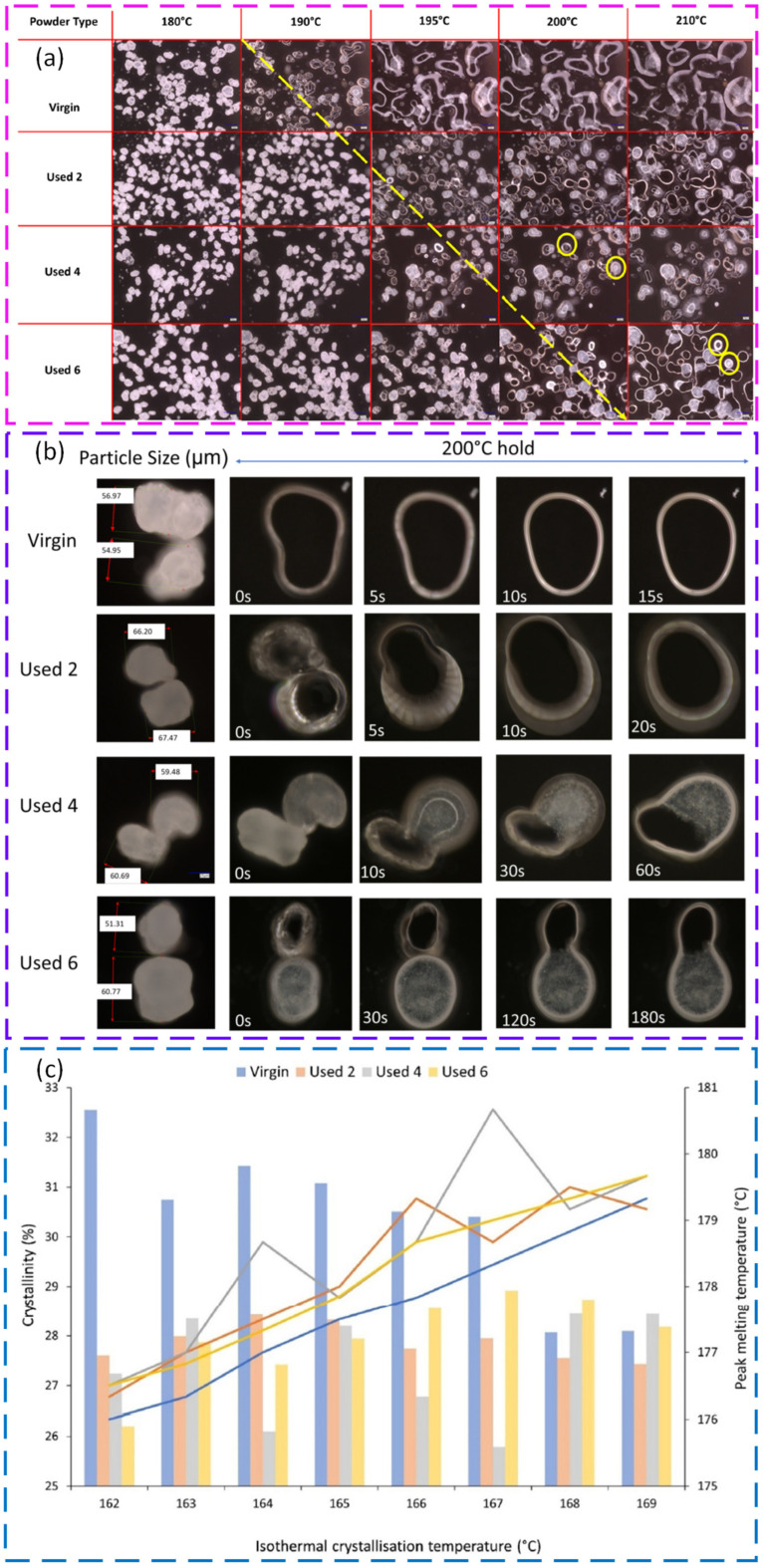
Effect of PA12 powder reuse in PBF: (**a**) hot stage microscopy during heating (10 °C/min) showing melting and coalescence of virgin and used powders (arrow marks softening→coalescence; circles highlight incomplete melting/unmelted cores); (**b**) particle coalescence at 200 °C; (**c**) crystallinity (bars) and peak melting temperature (lines) versus isothermal crystallisation temperature for virgin and used powders. Reproduced from [236], MDPI. 2024.

PA12 provides a detailed view of how reuse alters not only chemistry but coalescence and crystallisation kinetics under conditions relevant to production. Under an industrially relevant 70:30 refresh strategy (using virgin powder), repeated PBF exposure promoted polycondensation and crosslinking, increased molecular weight and glass transition temperature, and progressively reduced melt flow [236]. Hot stage microscopy showed that full particle coalescence at 200 °C slowed strongly from virgin to repeatedly reused powders, increasing the risk of incomplete melting and unmolten particle cores. Melting behaviour shifted and broadened, and isothermal crystallisation slowed. Crystallisation half-times increased, consistent with constrained chain mobility and hindered nucleation and growth, which also promotes surface defects such as “orange peel” (Figure 18) [236]. A particularly useful outcome is that reuse can invalidate simplified kinetic descriptions. In this study, the Avrami model no longer captured the full phase transformation of aged PA12, while the Hay model, which accounts for primary and secondary crystallisation, described the kinetics more accurately [236]. The broader implication is that sustainability strategies benefit from models that remain valid as the powder state evolves. Without this, refresh ratios risk becoming purely empirical and may drift out of the safe operating window as ageing progresses. 

Powder ageing, reuse, and refresh sit at the centre of a three-way trade-off between sustainability, process stability, and part quality in polymer PBF [233,234]. Reuse reduces waste but accumulated thermal and oxidative history can shift flowability and melt behaviour, narrow the process window, and increase defect probability. A defensible practice is therefore to couple refresh strategies with routine powder checks and simple go/no-go criteria for quarantine. Powder reuse should be communicated as state-based, not cycle-based. Decisions should be guided by rheology, DSC indicators, infrared indices, and process-relevant powder spreading checks, with clear thresholds for intervention. Post-processing should also be linked to dominant defect risks. Annealing or stress relaxation is most relevant when residual stresses and crystallinity gradients dominate, while infiltration or surface finishing is more relevant when internal voids and surface-connected porosity remain limiting. This lifecycle framing supports sustainability without sacrificing the repeatability required for qualification. 

## 8. PBF Application and Case Studies 

The case studies in this section are intentionally selective rather than exhaustive. They were chosen because they expose different failure modes and qualification constraints, and because they allow the powder to process to property logic to be traced with minimal ambiguity. In high impact polymer PBF, value is gained when an example does more than report a successful build. It should clarify which powder attributes mattered, which process levers stabilised powder spreading and laser coupling, and which acceptance metrics were decisive for the target function. These examples therefore act as worked references for designing experiments and for translating outcomes to qualification practice.

Polymer PBF is widely adopted where geometric freedom, short lead time, and material efficiency offer a clear advantage [237,238,239]. Typical outcomes include functional prototypes, housings with internal channels, lattice structures for energy absorption, and patient-specific components where conventional tooling is inefficient [240,241,242]. In healthcare, polymer PBF is attractive because anatomy-driven customisation can be paired with repeatable build workflows [240,241,242]. In pharmaceutical and drug-delivery contexts, porous architectures and controlled surface area can be used to tune release while maintaining compact device formats [243]. Across these domains, the same principle applies. Application success is rarely controlled by one parameter alone. It is controlled by the stability of powder spreading, the consistency of energy absorption, and the predictability of solidification or sintering under a defined thermal field.

A materials-driven example is provided by PEEK with nanocrystalline silicon nitride (PEEK/nSN), where bioresponse and process stability are pursued together [242]. In this study, low filler additions of 0.5 to 2.0 wt% nSN improved flow behaviour and reduced friction between particles, which supports more uniform layer formation. Crystallisation was also slowed, which is relevant because rapid crystallisation can amplify warpage risk in high temperature polymers. Optical response was improved, and the optimal laser power could be reduced, which helped limit thermal damage at high powers. A practical insight emerges. When an additive simultaneously improves powder handling and reduces the energy required for stable fusion, the process window can be widened in a way that remains useful for mechanical performance targets. In this case, diamond scaffolds with 1 wt% nSN showed the best balance, including higher modulus and yield strength than neat PEEK scaffolds, while remaining in a modulus range relevant for load sharing concepts [242].

**Figure 19:**
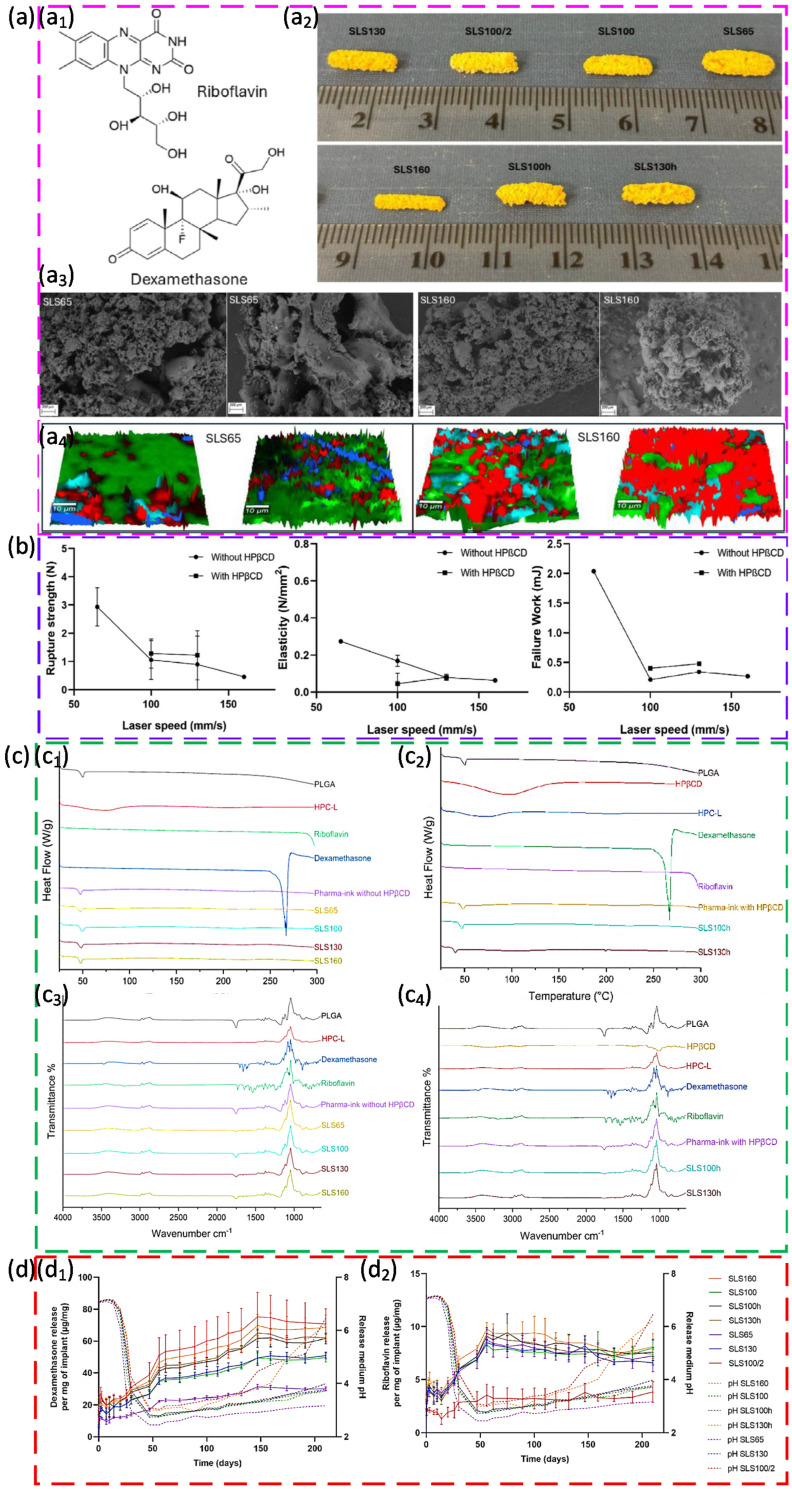
PBF drug-loaded intravitreal implants: (**a**) structural characterisation, including (**a1**) chemical structures of riboflavin and dexamethasone, (**a2**) printed implants, (**a3**) FE-SEM surface and cross-sections, and (**a4**) Raman maps of dexamethasone (red) and riboflavin (blue) at surface and core; (**b**) mechanical properties; (**c**) DSC and FTIR, including (**c1**) DSC without HPβCD, (**c2**) DSC with HPβCD, (**c3**) FTIR without HPβCD, and (**c4**) FTIR with HPβCD; (**d**) in vitro release and pH variation (pH 7.4), including early release (**d1**) dexamethasone and (**d2**) riboflavin over 7 days. Reproduced from [244], Wiley. 2025.

A second example shows how formulation design can be used to control both processability and long-term function in drug delivery. Biodegradable intravitreal implants were produced using PLGA-based powders with hydroxypropyl cellulose (HPC-L) and, in some formulations, hydroxypropyl β cyclodextrin (HPβCD) [244]. Processing was performed with a 445 nm laser and mild thermal conditions, while scanning speed was varied to adjust porosity and density without changing the drug chemistry. Chemical stability and homogeneous drug distribution were maintained and extended-release profiles were achieved by tuning the internal structure (see Figure 19). A key point for readers is that pharmaceutical PBF is not only a printing problem, it is also a powder and optical coupling problem. In this formulation, riboflavin acted as a photo-absorbing excipient and supported laser coupling, while the polymer matrix controlled the diffusion and release. This type of example is useful because it links composition, optical response, and final function within one controlled workflow [244].

**Figure 20:**
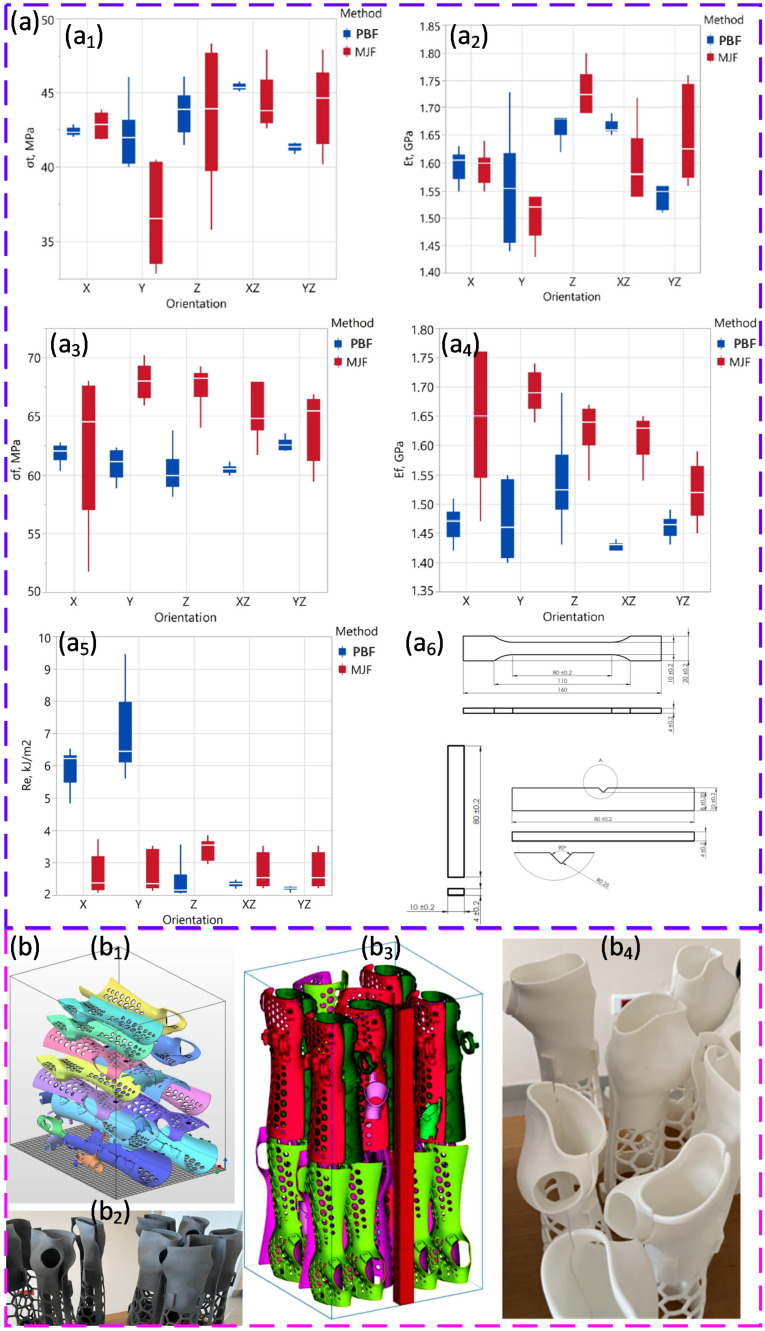
PBF and MJF forearm orthoses: (**a**) ISO-based mechanical properties versus build orientation, including (**a1**) ultimate tensile strength and (**a2**) Young’s modulus (ISO 527), (**a3**) flexural strength and (**a4**) flexural modulus (ISO 178), (**a5**) impact strength (ISO 179), and (**a6**) specimen dimensions; (**b**) case study of component alignment and printed orthoses, with (**b1**,**b2**) MJF and (**b3**,**b4**) PBF. Reproduced from [245], MDPI. 2024.

The next case study shifts from material function to medical device qualification and production planning. PA12 forearm orthoses were compared between PBF and HP Multi Jet Fusion (MJF), with testing across build orientations and with a production scenario based on hospital demand [245]. Mechanical differences were modest in tensile strength, while MJF showed more isotropic behaviour. Orientation effects were more visible in impact performance for PBF, which reinforces a practical message (see Figure 20). Qualification should include orientation sensitivity when patient safety and repeatability matter. A second practical message is that process throughput and cost are not independent of defect control. Packing density limits were required to avoid thermal defects, which shows that job-level thermal behaviour constrains production scaling even when the polymer is well established [245]. This example is valuable because it connects part performance, build orientation, and production constraints within one clinically motivated workflow.

Beyond biomedical parts, polymer PBF is increasingly used to deliver surface or bulk functionality that is difficult to obtain by post-processing alone. A clear example is the fabrication of bulk superhydrophobic components by embedding low-surface-energy PTFE grains inside thermoplastic matrices [246]. In this work, polypropylene with 22 wt% PTFE was processed under an optimised energy condition, and superhydrophobicity was retained even on fractured surfaces. This indicates a bulk effect rather than a fragile surface texture effect. Durability under abrasion and chemical exposure was also demonstrated. For readers, the key lesson is that robust wetting behaviour can be engineered into the bulk by controlling how functional domains are distributed and exposed during fusion, rather than relying on delicate microtextures that can be worn away [246].

A related functional direction is antimicrobial polymer composites. PA12 copper composites were produced by blending Cu into PA12-based powders for antibacterial applications [247]. Dense parts and homogeneous copper distribution were reported, and antibacterial activity was strong at higher Cu content. Mechanical behaviour showed the expected trade-off. Stiffness increased, while yield strength and ductility decreased as brittleness rose. This study provides a useful qualification-style message. Functional additives can deliver strong performance in the target domain, but the acceptance envelope must be defined explicitly. In antibacterial components, reduced ductility may be acceptable if the part is not load-critical, while density and surface integrity may remain strict requirements [247].

Electrical functionality and sensing provide another route to application-driven powder design. A conductive TPAE/MWCNT (thermoplastic polyether-block-amide elastomer/multiwalled carbon nanotube) composite powder was prepared to enable strain sensing and electro-induced shape memory behaviour [248]. A water-based preparation route produced a free-flowing powder, and conductive networks formed at particle interfaces after fusion. Electrical conductivity increased sharply near a percolation threshold, while mechanical performance peaked at moderate loadings and decreased at higher loadings due to defect formation and stress concentration effects. A practical pattern is repeated here. Functional percolation often arrives at the cost of process robustness if dispersion and interface bonding are not maintained. The study also demonstrates that PBF can deliver multifunctional elastomer composites when powder flow, dispersion quality, and fusion dynamics are balanced rather than maximised independently [248].

The case studies above also highlight a broader trend. Polymer PBF is moving from generic PA12 parts toward powders and processes tailored to specific functions. In bone tissue engineering, hydroxyapatite/polycaprolactone (HA/PCL) composite microspheres produced by a solid-in-oil-in-water route were engineered to be spherical, flowable, and thermally stable, so powder design, sintering window, and scaffold architecture could be tuned together [231]. Moderate HA contents of 5 to 10 wt% improved flow and mechanical reinforcement while supporting cell attachment and proliferation. Excessive filler loading disrupted coalescence and increased porosity, reducing mechanical integrity and biological response. This example is useful because it illustrates a repeatable design rule for composites. The filler level must be treated as a coupled variable that affects both powder spreading and fusion, not only the final modulus [231].

At the implant scale, the same powder-to-part logic was extended to patient-specific cranial plates by comparing neat PA12 with a carbon-filled composite (PA12CF35) in PBF, supported by thermomechanical finite element modelling [249]. The carbon-filled composite increased thermal conductivity and changed solidification behaviour, which reduced shrinkage and warpage while improving stiffness. Neat PA12 remained advantageous where compliance is needed. The main message is that composite selection can be used to steer the thermal field and distortion response, not only to change stiffness. This aligns with industrial qualification needs, where dimensional fidelity and residual stress risk can be just as decisive as strength [249].

Design guidance remains essential because geometry controls heat flow and stress development during printing. Rounded transitions, stable wall thickness where possible, and orientation choices that align critical features with more stable build directions can reduce distortion sensitivity. Drainage paths support powder removal from enclosed volumes, and nesting density affects job-level thermal uniformity. In practice, design choices act as thermal management tools as much as geometric choices. This view helps convert application targets into a stable build strategy rather than a search for a single “optimal” parameter set.

In summary, the strongest case studies do not only report that a part can be built, they clarify which powder attributes controlled the powder spreading and laser coupling, which process levers stabilised the window, and which defects were most limiting for the target function. The practical output for readers is a set of transferable patterns. Function often requires tailored chemistry or composites, but reliability still depends on a measurable powder state and controlled thermal history. This sets a clear bridge to the following section, where the remaining challenges and emerging directions are framed around metrology, lifecycle control, and predictive process maps. 

## 9. Challenges and Outlook

PBF of polymers inherits both its strengths and its limitations from the feedstock, and the central challenge is lot-to-lot consistency. Polymeric powders are highly sensitive to moisture, oxygen, and thermal history, so materials that look identical on a datasheet can spread and fuse very differently. Subtle shifts in particle size tails, shape, and surface chemistry alter packing, flowability, and melt behaviour, while conventional acceptance tests often compress rich distributions into single values that hide critical outliers. A more robust practice is to report distribution-level descriptors for size, shape, surface texture, and moisture sorption, and to link these directly to spreading quality and fusion behaviour through small, fast validation builds.

Powder production routes leave characteristic fingerprints in particle morphology, internal porosity, and surface residues. These features control thermal conductivity, porosity formation, cohesion, and tribocharging, and are difficult to hold constant when scaling from laboratory batches to industrial volumes. Progress depends on tighter control of solvents, surfactants, and stabilisers, combined with closed-loop feedback between detailed particle metrology and process parameters so that drifts are corrected before they appear on the build platform. Spreading and laser interaction add further variability: electrostatics and cohesion depend on humidity, temperature, fines content, and spreading tool design, while optical absorption at the laser wavelength is often adjusted with simple pigment additions that may age or disperse unevenly. Improved spreadability tests and sensorised intelligent powder spreaders [250] are expected to stabilise layer formation. More stable absorbers, such as nanoparticle additives [251] and carbon micro ballons [252], can also help. These absorbers can be chemically anchored to the powder surface or encapsulated. Together, these advances are expected to make the energy input more consistent.

**Figure 21:**
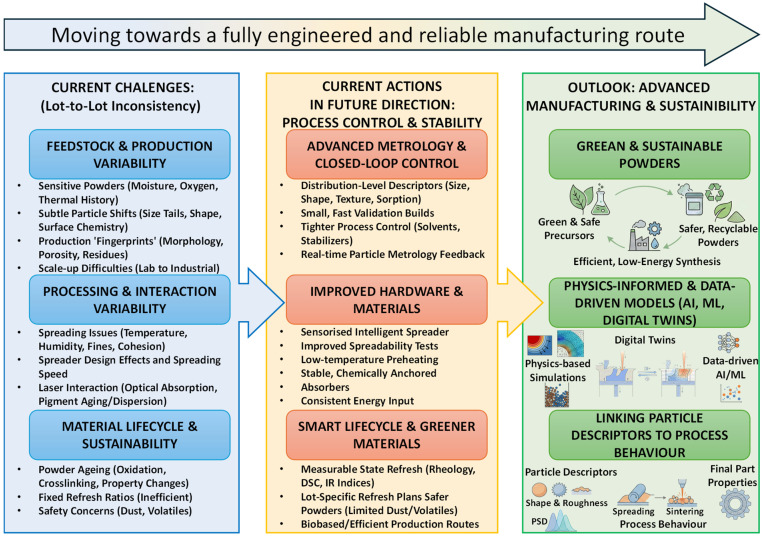
Roadmap toward an engineered polymer PBF route, summarising feedstock/processing variability, actions for metrology, and closed-loop control to stabilise powder spreading and laser coupling, and outlook themes covering lifecycle/sustainability and predictive modelling using physics-based simulations, AI/ML, and digital twins.

Powder ageing, reuse, and refresh strategies sit at the intersection of cost, quality, and sustainability. Long exposure to build temperatures can drive oxidation, chain scission, or crosslinking, change viscosity and crystallisation, and shrink the usable process window. Rather than relying on fixed refresh ratios, powders are increasingly treated as consumables with a measurable state, using rheology, DSC, infrared indices, and moisture uptake to design lot-specific refresh plans and to quarantine degraded material. In parallel, safer and greener powders are sought by limiting ultrafine dust and residual volatiles and by adopting more efficient, potentially bio-based production routes. Looking ahead, physics-informed and data-driven models, supported by AI, machine learning, and digital twins, are expected to link particle descriptors to spreading, sintering, and crystallisation, enabling predictive process maps. When combined with traceable, lot-aware workflows and adaptive refresh strategies, these developments will move polymeric PBF from empirical tuning toward a fully engineered and reliable manufacturing route (see Figure 21). 

## 10. Concluding Remarks

PBF of polymers performs best when powder, machine, and process are treated as a single engineered system. Among these, the powder is the least standardised, yet it largely determines what happens once the laser is applied. Particle chemistry controls melt viscosity, degradation, and crystallisation behaviour, while morphology and size distribution govern flow, packing, and local heat transfer. Surface texture and residuals influence cohesion, moisture uptake, and electrostatics. After spreading, these attributes shape the melt pool, the rate of neck growth, and the probability of lack of fusion, over-melting, or porosity.

Reliable practice therefore starts upstream. Each production route—such as cryogenic milling, spray drying, or polymerisation—imprints characteristic fingerprints on particle shape, internal porosity, and surface energy. Holding these within narrow limits, and reporting distribution-level data for size, shape, roughness, moisture sorption, and optical absorption at the laser wavelength, is more effective than relying on single acceptance numbers. Spreading then translates powder properties into bed quality: layer thickness, blade compliance, and speed, together with cohesion and tribocharging, must be tuned to achieve reproducible spread density, stable surface height, and minimal lift-off. Sensorised spreaders, non-contact spreaders, and benchtop tests that mimic real shear and contact conditions can support this tuning.

Thermal management, powder reuse, and standardisation complete the picture. Bed preheating, energy density, and scan strategy should be expressed as process maps, rather than as single “optimal” values, to reflect the thermal physics of semi-crystalline and amorphous polymers. At the same time, powder is best treated as a monitored consumable, with rheology and thermal analysis guiding refresh ratios and quarantine. In the long term, standardised descriptors, data-driven models, and lot-aware workflows will turn polymer powders from uncertain inputs into designed, traceable components of a robust PBF process.

## Figures and Tables

**Table 1:** Reporting and Process Window Checklist for PBF of polymers.

Material	Powder Descriptors (PSD and Morphology)	Layer Thickness	Hatch Spacing/Distance	Scan Speed and Laser Power	Bed/Chamber Temperature	Atmosphere	Reuse/Refresh Protocol	Ref.
Polyamide 6 (PA6)	Spherical; average particle size 58.9 μm; Gaussian distribution.	100–120 µm.	0.3 mm.	400, 3500 mm/s; 7, 15–30 W.	Bed: 140, 195 °C.	Nitrogen protecting atmosphere.	Virgin vs. 100% reused.	[88,225]
Polyamide 11 (PA11)	~45 µm average size. Near-spherical.	100 µm.	0.25 mm- 0.09 (semi-sintering); 0.04 (melting).	Scan speed Not reported but build rate: 0.0033 mm/s; 10,000 mm/s (semi-sintering); 4000 mm/s (melting Power: 27 W; 4–15 W.	Bed: 187 °C; 23–190 °C while 150 °C (low-temp process to avoid ageing) Sintering window: 170–190 °C.	Argon recommended or Purged with Nitrogen.	Studied up to 10 cycles with 100% reused powder.	[85,86,226]
Polyamide 12 (PA12)	PSD: 20–80 µm recommended. Average particle size 53.8 μm Near-spherical “potato” shape.	100–150 µm. Recommended to be at least 2× the average particle size.	0.22–0.3 mm.	Speed: 2500–4000 mm/s. Power: 18–21 W or 75–95% of machine max.	Bed: 167–170 °C. Chamber: 135 °C.	Air or Argon or Nitrogen.	30:70 to 50:50 ratio (Virgin: Used). Virgin vs. 100% reused. 70% refresh ratio commonly used.	[55,214,225,227]
PEEK (Polyetheretherketone)	Average ~75 µm. d_10_: 50 µm; d_90_: 108 µm. Morphology: Irregular and least spherical.	80–100 µm.	0.1–0.25 mm.	Speed: 2000–3000 mm/s. Power: 6–36 W depending on layer and recycle status.	Bed: 313 °C. Sintering window: 297.9–322.6 °C.	Argon (Ar) is highly recommended for maintaining hardness. Nitrogen (N) is acceptable but reduces hardness after 4 h of ageing. Air causes severe blackening and degradation.	Potential for 100% aged powder reuse in inert atmosphere.	[97,99,228,229,230]
PEKK (Polyetherketoneketone	Broad distribution. D10: 26 µm, D50: 52.8 µm, D90: 181.6 µm. Morphology: Heterogeneous and porous.	120 µm.	Hatching energy density: 23.5 mJ/mm^2^. Beam offset: 0.39 mm.	Speed: 1000 mm/s Power: 8.5 W.	292 °C	Inert (standard for PAEK family).	Information not detailed in sources.	[98]
PPS (Polyphenylene Sulfide)	Average ~75 µm.	102 µm.	0.1524–0.2286 mm.	Speed: 2000–3000 mm/s. Power: 12–18 W.	Bed: 240 °C.	Inert environment suggested.	Information not detailed in sources.	[204]
TPU (Thermoplastic Polyurethane)	20–105 µm granulation. Near-spherical.	75–200 µm.	0.075–0.1 mm.	Speed: 7600 mm/s. Power: 60 W or Laser Power Ratio 1.0–2.0.	Bed: 95 °C.	Not specified (often ambient or low preheat).	0% refresh ratio (100% reusable) for Flexa Black.	[105,107]
Polypropylene	Mean: 90–158 µm across reuse cycles. Irregular morphology.	150 µm. Should be ~300 µm for this PSD.	0.25 mm.	Speed: 4500 mm/s. Power: 35 W (Fill).	Bed: 128 °C. Removal chamber: 125 °C.	Information not detailed in sources.	100% re-usable for 5 cycles without virgin mixing.	[96]
PET (Polyethylene Terephthalate)	Experimental grade from SABIC.	100 µm.	Information not detailed in sources.	Speed: 90 mm/s Power: 100% lamp power.	Process: 190–228 °C.	Information not detailed in sources.	100% virgin ratio typically reported.	[92]
HDPE (High-Density Polyethylene)	Average: 69.62 µm.	100 µm.	0.25 mm.	Energy density: 4.53–9.07 J/m.	Bed: 120–125 °C. Chamber: 120 °C.	Information not detailed in sources.	Information not detailed in sources.	[93]
PCL (Polycaprolactone)	Spherical with some wrinkles/micropores; D50 98 μm.	100 µm	0.1 mm.	3600 mm/s; 2.8–5.6 W.	Bed: 50 °C	Nitrogen atmosphere.	Not explicitly detailed	[231]
PCL/HA (PCL/Hydroxyapatite)	Spherical; HA uniformly distributed on surface; D50 ~70 μm.	100 µm	0.1 mm.	2400–3600 mm/s; 2.8–7.0 W.	Bed: 54–58 °C	Nitrogen atmosphere.	Not explicitly detailed	[231]
PA12-CF (Carbon Fiber reinforced)	PA12 particles < 100 µm; CF rod-shaped (80 µm length).	100 µm	Single-window, constant-time pattern.	400–2000 mm/s (multi-pass); 5 W.	Bed: 150–162 °C	Ambient air.	Only fresh (unused) powder used to ensure consistency	[232]
PA12/PF (Thermoset Composite)	PF: 60.5 µm; PA12: 58.0 µm.	200 µm	0.15 mm.	Speed: 100–250 mm/s. Power: 7–9 W.	Bed: 70 °C.	Information not detailed in sources.	Information not detailed in sources.	[47]

## Data Availability

No new data were created or analyzed in this study. Data sharing is not applicable to this article.
